# The Whole-transcriptome Landscape of Diabetes-related Sarcopenia Reveals the Specific Function of Novel lncRNA Gm20743

**DOI:** 10.1038/s42003-022-03728-8

**Published:** 2022-08-01

**Authors:** Jing Yu, Kim Loh, He-qin Yang, Meng-ran Du, Yong-xin Wu, Zhi-yin Liao, Ai Guo, Yun-fei Yang, Bo Chen, Yu-xing Zhao, Jin-liang Chen, Jing Zhou, Yue Sun, Qian Xiao

**Affiliations:** 1 Department of Geriatrics, The First Affiliated Hospital of Chongqing Medical University, Chongqing, China; 2 Diabetes & Metabolic Disease Laboratory, St. Vincent’s Institute of Medical Research, Fitzroy, Melbourne, VIC Australia; 3 Health Outcome Research and Policy, Harrison School of Pharmacy, Auburn University, Auburn, AL USA; 4 Department of Neurosurgery, The First Affiliated Hospital of Chongqing Medical University, Chongqing, China; 5 Department of Anesthesiology, Chongqing University Cancer Hospital, Chongqing, China; 6 Department of Clinical Medicine, Chongqing Medical and Pharmaceutical College, Chongqing, China

**Keywords:** Diabetes complications, Long non-coding RNAs

## Abstract

While the exact mechanism remains unclear, type 2 diabetes mellitus increases the risk of sarcopenia which is characterized by decreased muscle mass, strength, and function. Whole-transcriptome RNA sequencing and informatics were performed on the diabetes-induced sarcopenia model of *db/db* mice. To determine the specific function of lncRNA *Gm20743*, the detection of Mito-Sox, reactive oxygen species, Ethynyl-2′-deoxyuridine, and myosin heavy chain was performed in overexpressed and knockdown-*Gm20743* C2C12 cells. RNA-seq data and informatics revealed the key lncRNA-mRNA interactions and indicated a potential regulatory role of lncRNAs. We characterized three core candidate lncRNAs *Gm20743, Gm35438, 1700047G03Rik*, and their potential function. Furthermore, the results suggested lncRNA *Gm20743* may be involved in regulating mitochondrial function, oxidative stress, cell proliferation, and myotube differentiation in skeletal muscle cells. These findings significantly improve our understanding of lncRNAs that may mediate muscle mass, strength, and function in diabetes and represent potential therapeutic targets for diabetes-induced sarcopenia.

## Abstract (web summary)

The role of lncRNA Gm20743 in the development of diabetic sarcopenia is explored using a mouse model.

## Introduction

Obesity and diabetes are serious global health problems. Obesity is a global chronic health problem attributed to almost 44% of type 2 diabetes mellitus (T2DM)^1,2^. Skeletal muscle is a major organ for glucose metabolism. In addition, skeletal muscle loss is closely related to insulin resistance and metabolic syndromes such as obesity and diabetes^3^. Loss of skeletal muscle and intramuscular fat disposition is associated with various pathological mechanisms, including insulin resistance, oxidative stress, mitochondrial dysfunction, and inflammatory cytokines^4^. As a chronic complication of diabetes, the progress of sarcopenia is further exacerbated in patients with T2DM and is a serious risk factor that may result in mortality^5^. Sarcopenia is characterized by a loss of skeletal muscle strength, mass, and physical function^6^. According to a recent review in The Lancet (2019), sarcopenia can occur during mid-life and is associated with a range of conditions such as obesity and T2DM.^7^. Decreased muscle strength, mass, and physical function were reported in T2DM patients compared to healthy individuals^8,9^. Rodent research also found that the accumulation of advanced glycation end-products may impair muscle mass and function, leading to muscle loss in diabetic mice^10^. Moreover, with a higher risk of falls, disability, and mortality, sarcopenia associated with obesity and diabetes impairs the individual’s quality of life and the prognosis of T2DM^11,12^. Although there are many known risk factors and symptoms of diabetes-associated sarcopenia, the underlying mechanism remains unclear.

Most of the human genome is expressed as primary transcripts. However, only approximately 2% of transcripts (known as messenger RNAs, mRNAs) encode functional proteins, while non-coding transcripts (known as non-coding RNAs, ncRNAs) account for approximately 98% of transcripts^13^. NcRNAs are mainly divided into two categories: long non-coding RNAs (lncRNAs) and microRNAs^14^. LncRNAs are a class of longer than 200nt RNAs with weak or no protein-coding potential^15^. LncRNAs have a lower transcript abundance but show stronger tissue specificity^16^. LncRNAs can bind to microRNA sites as competing endogenous RNAs, and the co-expression of lncRNA-mRNA is involved in pathological and physiological functions in skeletal muscle cells^17^. LncRNAs are closely related to the progression of skeletal muscle and diabetes development^18^, serving as therapeutic targets, potential biomarkers, or indicators of prognosis. However, the gene expression profiling and precise regulatory roles of lncRNAs during diabetes-induced sarcopenia are still unknown.

In our study, we utilized an overt obese and diabetes *db/db* mice model in which muscle loss was evidenced and an acute stimulation in vitro model of palmitic acid (PA)-treatment in C2C12 myotubes. Skeletal muscle fibres can be categorized into slow (Type I) and fast-twitch fibres (Type II)^19^, the preferred atrophy of type II fast-twitch fibres like gastrocnemius (GAS) seems to occur in patients with diabetes and muscle atrophy^20^. Therefore, whole-transcriptome sequencing was performed on the GAS from *db/db* mice and their littermates *db/m* mice. Subsequently, differential expression and functional prediction were performed to explore the gene-regulatory circuits that are associated with diabetes-related sarcopenia. In addition, the candidate lncRNAs (such as *Gm20743* (log2FC = −1.57, *q*-value < 0.001), *Gm35438* (log2FC = −1.90, *q*-value < 0.001) and *1700047G03Rik* (log2FC = 6.34, *q*-value < 0.001)) and their potential function were identified by co-expression network and informatics. In this study, we will focus on the specific function of lncRNA *Gm20743* in the development of diabetes-related sarcopenia. An in vitro cultured skeletal muscle cells with overexpressed and knockdown *Gm20743* were applied to further determine the role of *Gm20743* in regulating mitochondrial function, oxidative stress, cell proliferation, and myotube differentiation. Together, this study provides novel insights into the regulatory molecular mechanism of the progression of diabetes-related sarcopenia and potential targets for early diagnosis and therapeutic implications.

## Results

### Model-identification of diabetes-related sarcopenia in *db/db* mouse

**Fig. 1:**
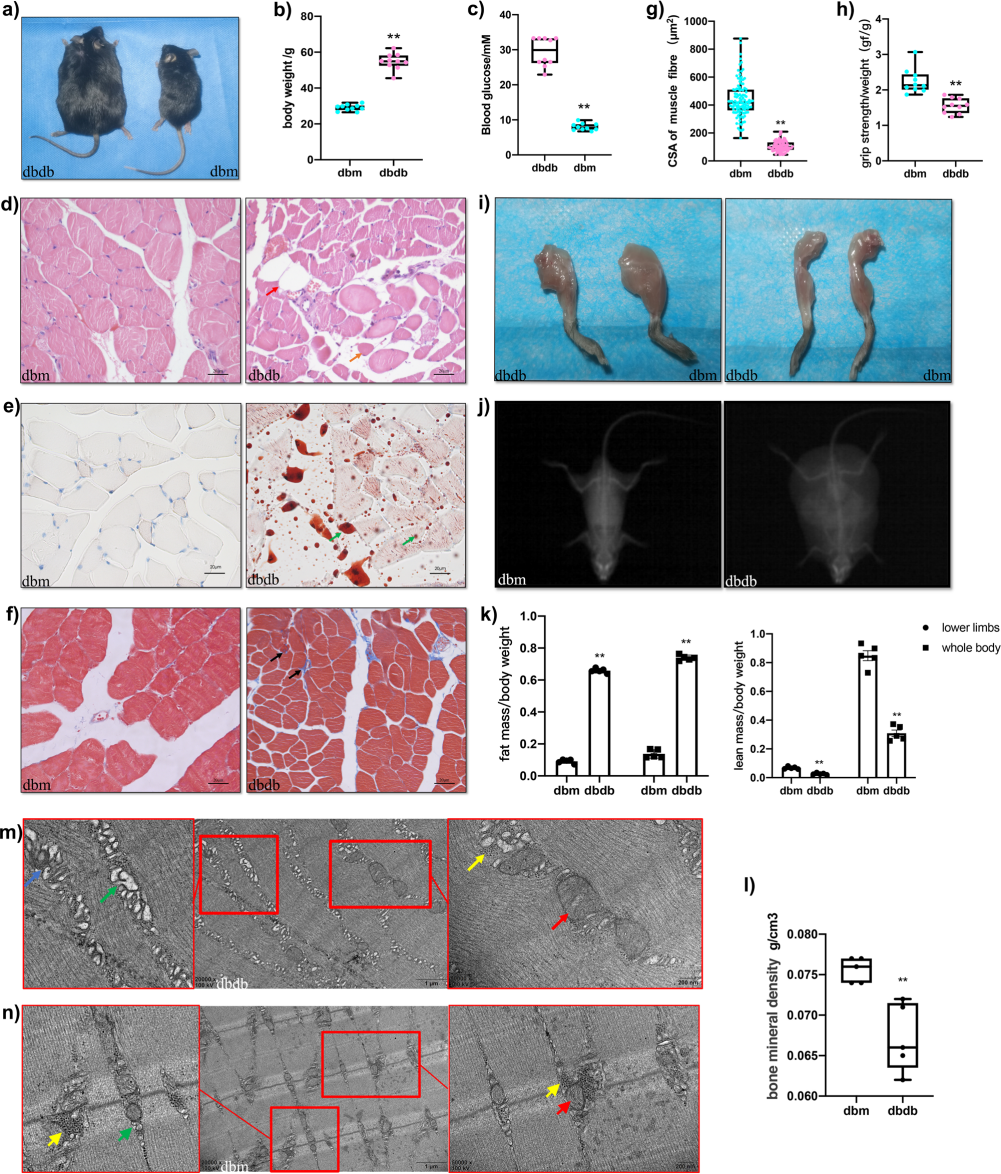
A metabolic disorder, loss of muscle mass and strength, and structural abnormalities are evident in the diabetic muscle atrophy model of *db/db* mice. Compared to *db/m* group, *db/db* mice extremely developed obesity (**a**, **b**), hyperglycaemia (**c**) (*n* = 10). H&E (**d**), Oil-red O staining (**e**), and Masson staining (**f**) in GAS of *db/db* and *db/m* group. Scale bar, 20 μM. Red arrow indicates adipocytes; orange arrow indicates atrophic myofibres; green arrows indicate lipid droplets; black arrows indicate muscle fibrosis. **g** Quantitative analysis of the average cross-sectional area (CSA) of myofibres. **h** The forelimb grasping strength and **i** muscle mass and size of lower limbs were decreased in *db/db* mice compared with *db/m* mice. The whole-body composition analysis of dual‐energy X‐ray absorptiometry (DEXA) (**j**) displayed more fat mass, less lean mass (**k**) and decreased bone mineral density (BMD) (**l**) in *db/db* vs. *db/m* (*n* = 5 replicates). Transmission electron microscopy (TEM) of GAS from *db/db* and *db/m* mice (**m**, **n**). The long red arrow indicates mitochondrial abnormalities and the short one indicates normal mitochondria; the blue arrow indicates myelinbody; the yellow arrows indicate muscular glycogen; the long green arrow indicates swelling endoplasmic reticulum and the short one indicates normal endoplasmic reticulum. Data were expressed as mean ± S. D. *P < 0.05 **P < 0.01. Note: H&E: haematoxylin and eosin; GAS: gastrocnemius; CSA: average cross-sectional area; DEXA: dual‐energy X‐ray absorptiometry; BMD: decreased bone mineral density; TEM: Transmission electron microscopy.

Fifteen-week-old *db/db* mice showed a phenotype of severe obesity (Fig. 1a, b) with obvious hyperglycaemia (>25 mmol/L) (Fig. 1c) compared to normal control *db/m*. Morphological changes in GAS were observed by haematoxylin and eosin (H&E), Oil-Red O, and Masson’s trichrome staining compared to *db/m mice* (Fig. 1d–f). The average cross-sectional area (CSA) of muscle fibres was markedly reduced (Fig. 1g), and lipid droplet infiltration and increased fibrosis were observed in GAS of *db/db* mice, indicating evident pathological changes of diabetes-induced sarcopenia. The forelimb grip strength of *db/db* mice was significantly decreased (Fig. 1h), and the muscle mass and size of lower limbs were decreased (Fig. 1i) compared with *db/m* mice. These results indicate that muscle strength and mass were significantly decreased in *db/db* mice. Decreased bone mineral density (BMD), lower lean mass, and increased fat mass were observed by dual‐energy X‐ray absorptiometry (DEXA, Hologic Discovery A, Hologic Inc) analysis in the *db/db* group (Fig. 1j–l). Serum biochemical parameters were indicative of the systematic metabolic disorder. Triglycerides, glucose, total cholesterol, low-density lipoprotein cholesterol, and high-density lipoprotein cholesterol were increased significantly in *db/db* mice compared to *db/m* control (Supplementary Table 1). Morphological abnormalities of mitochondria, swelling of the endoplasmic reticulum, reduced muscular glycogen, and sarcomere damage were observed by transmission electron microscopy (TEM) in GAS of *db/db* compared with the *db/m* group (Fig. 1m, n). Our data showed that muscular weakness and loss with subcellular structural dysfunction are evident in *db/db* mice.

### Identification of dif-mRNAs in GAS of *db/db* mice

**Fig. 2:**
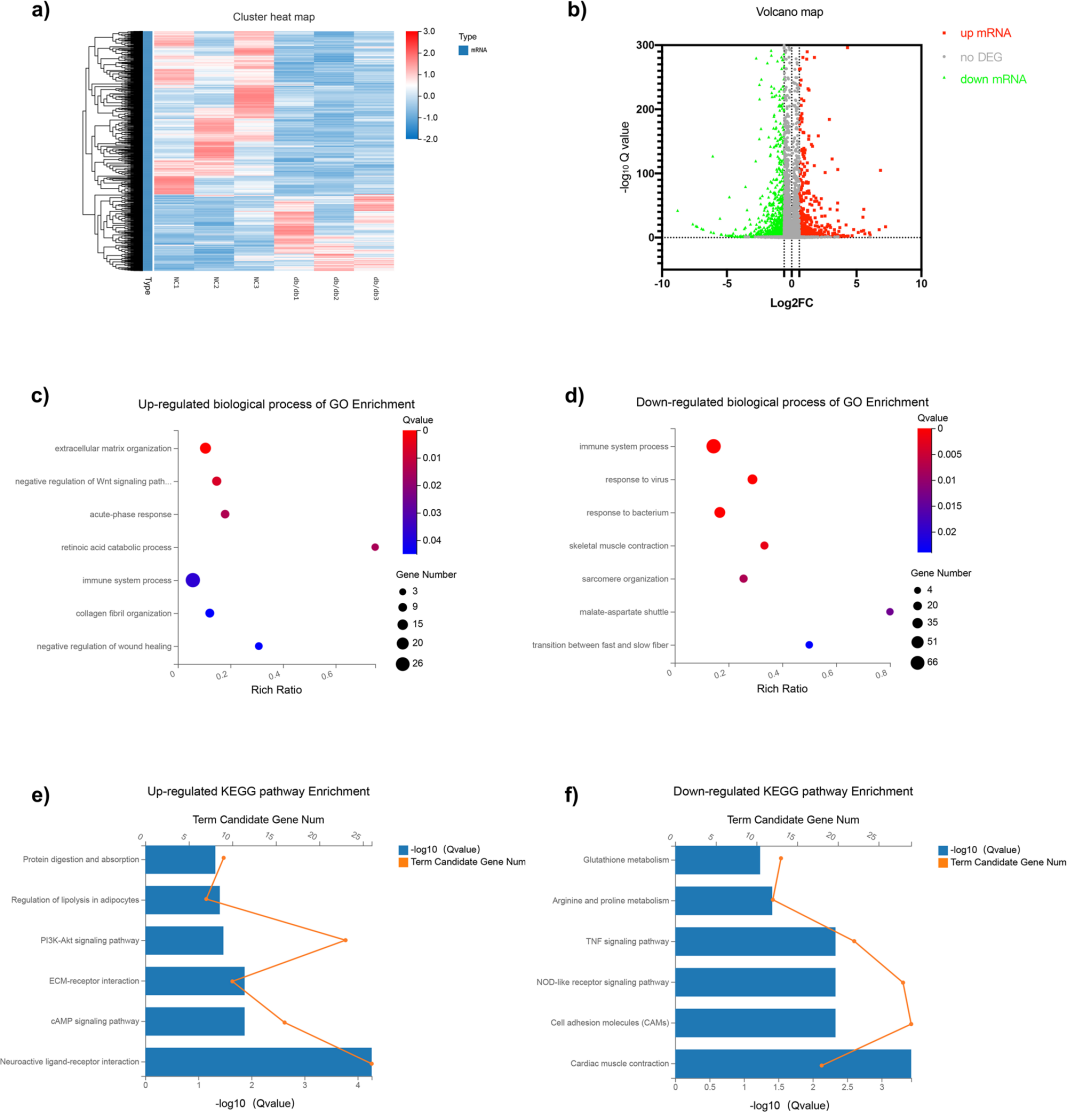
RNA sequencing profile and functional enrichment analysis of DEGs. **a** Hierarchical clustering of mRNA sequencing showed mRNA differential expression profiles (|log_2_ FC | < 0.585, FDR < 0.05) of GAS between *db/db* and *db/m* (NC) group. Each row indicates a single gene, and each column indicates the GAS tissue of a mouse. **b** The scatter plot was performed for the alteration of dif-mRNAs in GAS of *db/db* vs. *db/m* (*n* = 3). Red plots represent up-regulated mRNAs (log_2_ FC > 0.585, FDR < 0.05) and green plots represent down-regulated genes (log_2_ FC < −0.585, FDR < 0.05). The significant GO terms (q-value < 0.05) of mRNA expression variation between two compared groups were listed (**c**, **d**). The top significant KEGG pathway terms (q-value < 0.05) of dif-mRNAs were listed (**e**, **f**). Note: NC: normal control group. GAS: gastrocnemius; dif-mRNAs: differentially expressed mRNAs; DEGs: differential expression of genes; FC: fold change; FDR: false discovery rate; GO: Gene Ontology; KEGG: Kyoto Encyclopedia of Genes and Genomes.

To identify new molecular mechanisms during diabetes-accelerated sarcopenia, total RNA from GAS of *db/db* and *db/m* mice was used for whole transcriptome sequencing. Raw sequencing data were filtered, and clean reads were mapped to the reference genome (reference species: Mus_musculus. Reference Genome Version: GCF_000001635.26_GRCm38.p6). Filtered clean reads were spliced into putative transcripts (Supplementary Table 2). An absolute log_2_ FC value of 0.585 was used as the standard to confirm the dif-mRNAs (false discovery rate, FDR < 0.05). Based on that standard, 607 upregulated mRNAs and 1332 downregulated mRNAs were identified in *db/db* vs. *db/m* (Supplementary Data 1–2). The heatmap and a volcano plot (Fig. 2a, b) showed differential mRNA expression between *db/db* vs. *db/m* (FDR < 0.05). The top 20 dysregulated mRNAs are shown in Supplementary Table 3. The top5 up-regulated mRNAs are *Ttll7, Sorbs2, Sh3rf2, Prelp*, and *Ppp1r3c*, while the top5 down-regulated genes include *Xirp1, Vldlr, Vgll2, Ugp2*, and *Ucp3* in Supplementary Table 3.

### Functional prediction of differentially expressed mRNAs (dif-mRNAs)

Gene Ontology (GO) and Kyoto Encyclopedia of Genes and Genomes (KEGG) pathway enrichment analyses were performed to identify potential biological functions and implications. Dif-mRNAs were exhibited in three modules of the GO pathway enrichment analysis in GAS of the *db/db* group. Upregulated mRNAs were mainly enriched in the extracellular matrix (ECM) organization (GO:0030198), negative regulation of the Wnt pathway (GO:0030178), retinoic acid catabolic process (GO:0034653), inflammatory response (GO:0006954), and collagen fibril organization (GO:0030199) in the biological process category of GO terms (Fig. 2c). Downregulated mRNAs were primarily enriched in terms such as immune system process (GO:0002376), and skeletal muscle contraction (GO:0006936) (Fig. 2d). The top 10 GO terms associated with dif-mRNAs of *db/db* mice are shown in Supplementary Table 4–5. For dif-mRNAs, enriched transcription factors (TFs), including *Nr4a1, Mafa, Jun* (also known as *AP-1*), *ATF5, Trp63*, and *Tbx1*, were differentially expressed in the *db/db* group (Supplementary Table 6). These data showed that differential TFs might be essential for the progression of diabetes-induced sarcopenia.

Then, 607 upregulated mRNAs and 1332 downregulated mRNAs were enriched in dysregulated pathways by KEGG analysis. Our data showed that 607 upregulated genes were enriched in 204 signalling pathways, which were mainly enriched in the pathway of neuroactive ligand-receptor interaction (PATH: map04080), cAMP signaling (PATH: map04024), ECM-receptor interaction (PATH: map04512), regulation lipolysis in adipocytes (PATH: map04923) and protein digestion and absorption (PATH: map04974) (Fig. 2e). KEGG enrichment showed that 1332 downregulated genes were enriched in 249 significant pathways, including the pathway of muscle contraction (PATH: map04260), cell adhesion molecules (PATH: map04514), TNF signaling (PATH: map04668), arginine and proline metabolism (PATH: map00330), and glutathione metabolism (PATH: map00480) (Fig. 2f). The top 10 KEGG pathways associated with dysregulated mRNAs of diabetes-related sarcopenia are shown in Supplementary Table 7–8, indicating that these significant changes in pathways may contribute to the development of diabetes-induced sarcopenia.

Based on shared biological function, location, or expression of genes, Gene set enrichment analysis (GSEA) has a broad scope of enrichment analysis and discovers potential mechanisms in disease. The dif-mRNAs were associated with significant pathways in GSEA, as shown in Supplementary Table 9 (*p*-value < 0.05). The GSEA results showed that the top 2 upregulated mRNAs were closely related to neuroactive ligand-receptor interactions (PATH: map04080) and the renin secretion pathway (PATH: map04924) (Supplementary Fig. 1a, b). In contrast, the top 2 downregulated mRNAs were highly correlated with the pathway of oxidative phosphorylation (PATH: map00190), arginine and proline metabolism (PATH: map00330) (Supplementary Fig. 1c, d).

### Pathway-act-network

To investigate deeper interactions in significant pathways, 370 dif-mRNAs enriched into 10 mainly significant pathways of KEGG were subjected to a visual pathway-act-network analysis (Supplementary Fig. 2a). A pathway-act network was constructed with 233 dif-mRNAs enriched with 10 significant pathways of GSEA (Supplementary Fig. 2b). These data showed that significant metabolic pathway regulation might be involved in the development of diabetes-induced sarcopenia.

### Identification of differentially expressed lncRNAs (dif-lncRNAs) in GAS of *db/db* vs. *db/m*

**Fig. 3:**
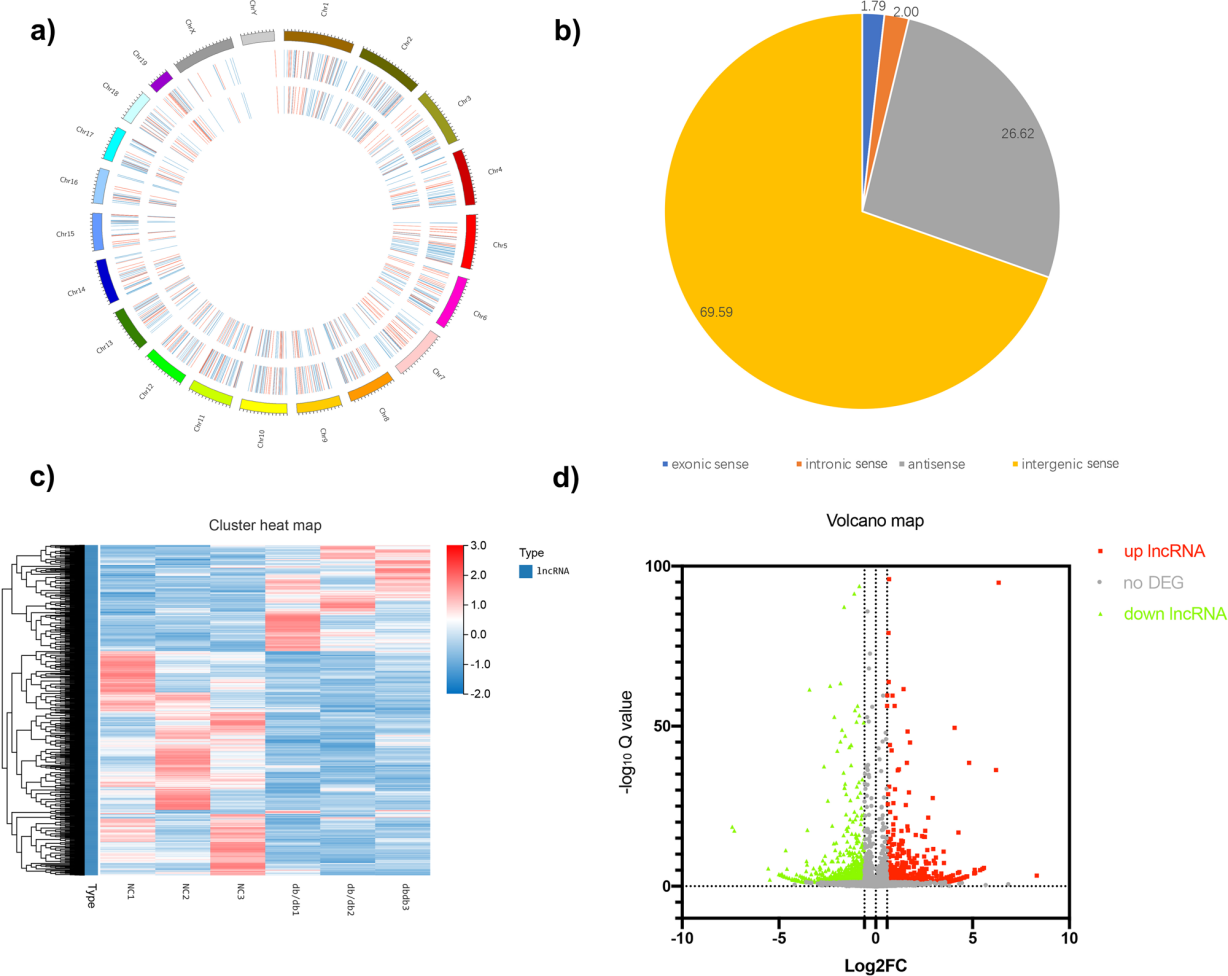
Differentially expressed lncRNAs in *db/db* vs. *db/m*. **a** Circus plots showed dif-lncRNAs on mouse chromosomes for *db/db* vs. *db/m*. The outer circle is the chromosome, the second circle is all the differential dysregulated lncRNAs, and the inner circle is the significantly dif-lncRNAs (|log_2_ FC | > 1 q-value < 0.05). **b** Pie charts showed the percentage of four types (exonic sense, intronic sense, antisense, intergenic sense) of dysregulated lncRNAs in *db/db* mice and the controls. **c** The cluster heatmaps and **d** scatter plots showing the dif-lncRNAs in *db/db* and *db/m* mice, respectively (*n* = 3). Note: dif-lncRNAs: differentially expressed lncRNAs.

According to the screening criteria, 306 upregulated lncRNAs and 554 downregulated lncRNAs were identified in *db/db* vs. *db/m* (Supplementary Data 3–4). All dif-lncRNAs were widely found on all chromosomes, including the X and Y sex chromosomes (Fig. 3a). These dif-lncRNAs were divided into four categories based on different genic loci: exonic sense, intronic sense, antisense, and intergenic (Fig. 3b). Heatmap and volcano plot were constructed (Fig. 3c, d). We also identified the top 20 dysregulated lncRNAs in *db/db* mice (Supplementary Table 10).

### Co-expression network construction and Real-Time Quantitative PCR (RT-qPCR) validation

**Fig. 4:**
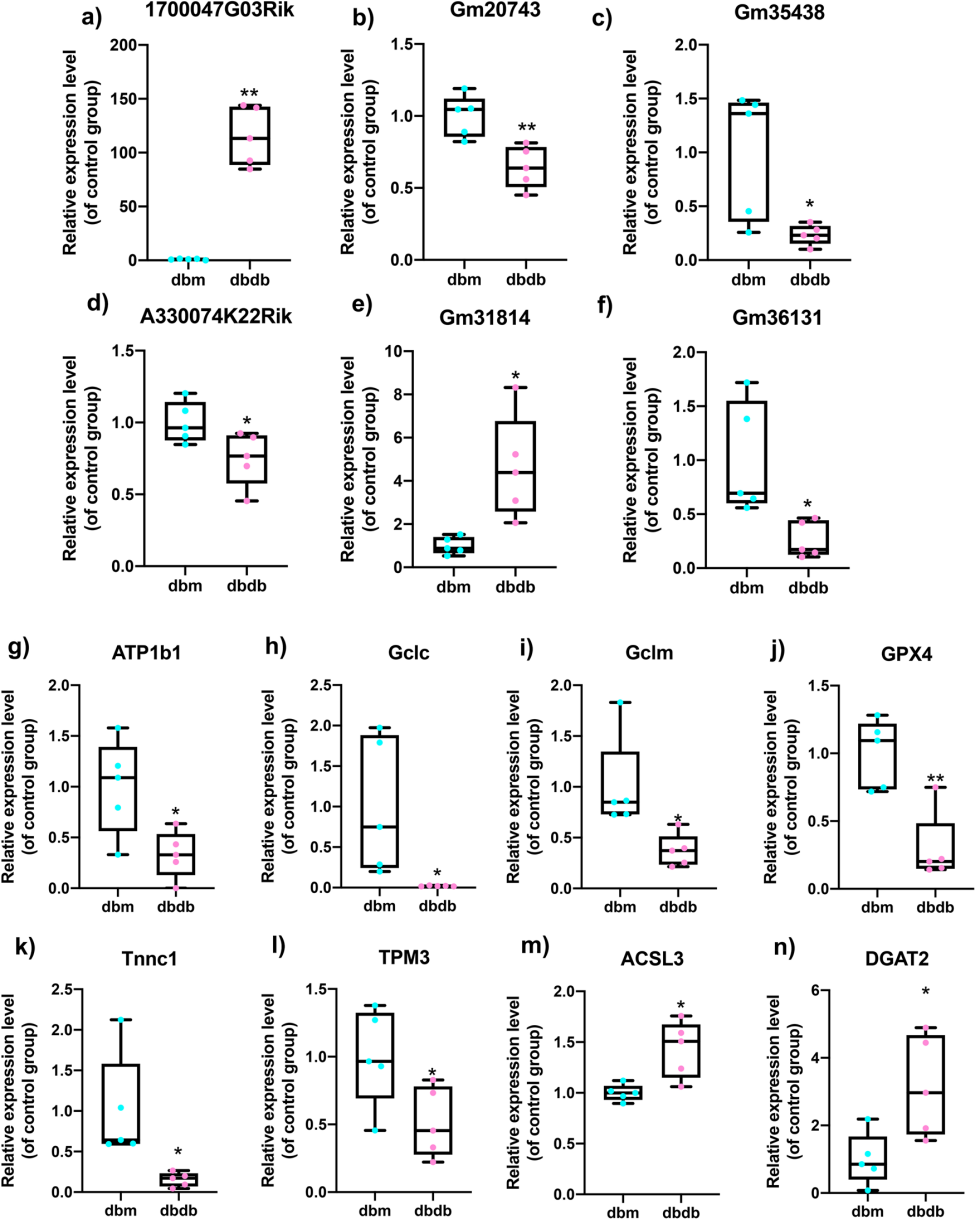
Validation of the expression of dif-lncRNAs and dif-mRNAs in *db/db* and *db/m* mice. The relative expression of dif-lncRNAs (|log2 FC | > 1, expression >2 and *q*-value <0.01) was detected by RT-qPCR in GAS of *db/db and db/m* mice (**a**–**f**). The relative expression of closely related dif-mRNAs involved in significant pathways was detected in GAS of *db/db and db/m* mice (**g**–**n**) (*n* = 5). Data were expressed as mean ± S.D. *P < 0.05 **P < 0.01. Note: RT-qPCR: Real-Time Quantitative PCR.

**Fig. 5:**
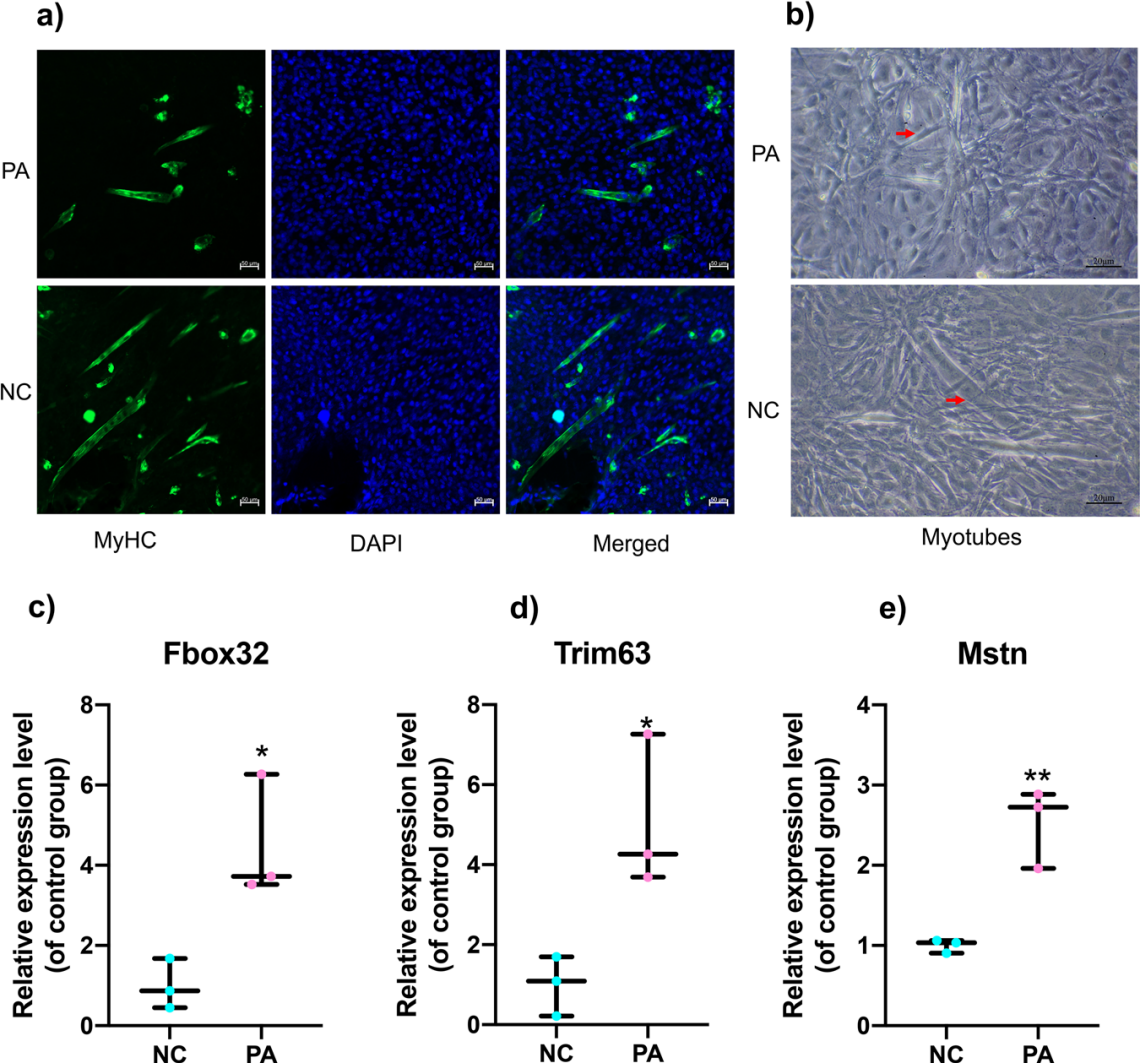
PA-induced muscle atrophy model in C2C12 cells. The decreased CSA and numbers of myotubes were observed in PA-treated C2C12 cells through fluorescently labeled MyHC by confocal microscopy (**a**) and light microscopy (**b**). The higher expression of Fbox32 (**c**), Trim63 (**d**), Mstn (**e**) suggested that PA-induced muscle atrophy in C2C12 cells. Data were expressed as mean ± S.D. *P < 0.05 **P < 0.01. Note: PA: palmitic acid; MyHC: myosin heavy chain.

**Fig. 6:**
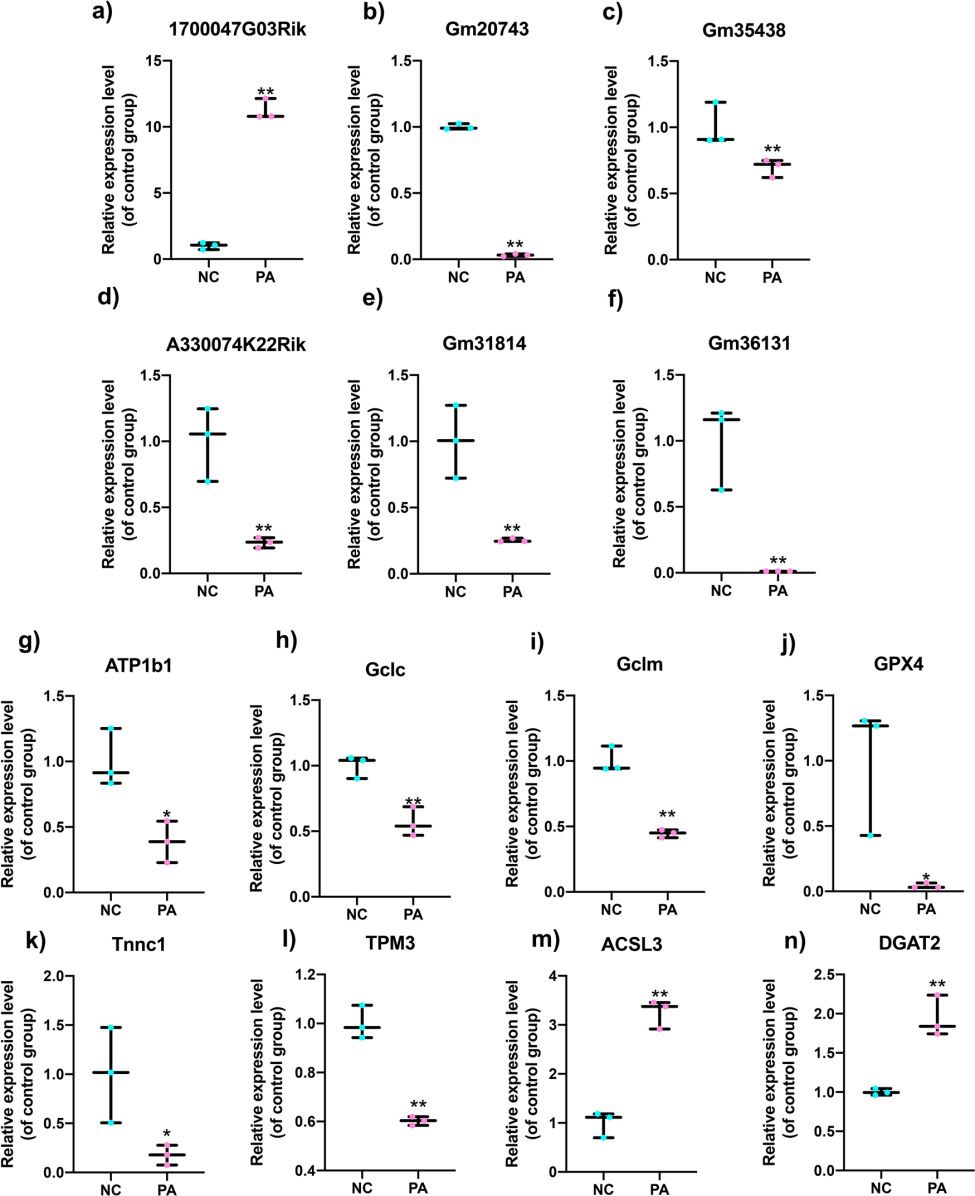
Validation of the expression of dif-lncRNAs and dif-mRNAs in C2C12 cells exposed to PA. The relative expression of dif-lncRNAs (|log2 FC | > 1, expression >2 and q-value < 0.01) was detected in PA-induced C2C12 cells compared with NC group (**a**–**f**). The relative expression of highly correlated dif-mRNAs involved in the co-expression network was detected in PA-induced C2C12 cells and the control group (**g**–**n**). Data were expressed as mean ± S.D. *P < 0.05 **P < 0.01.

**Fig. 7:**
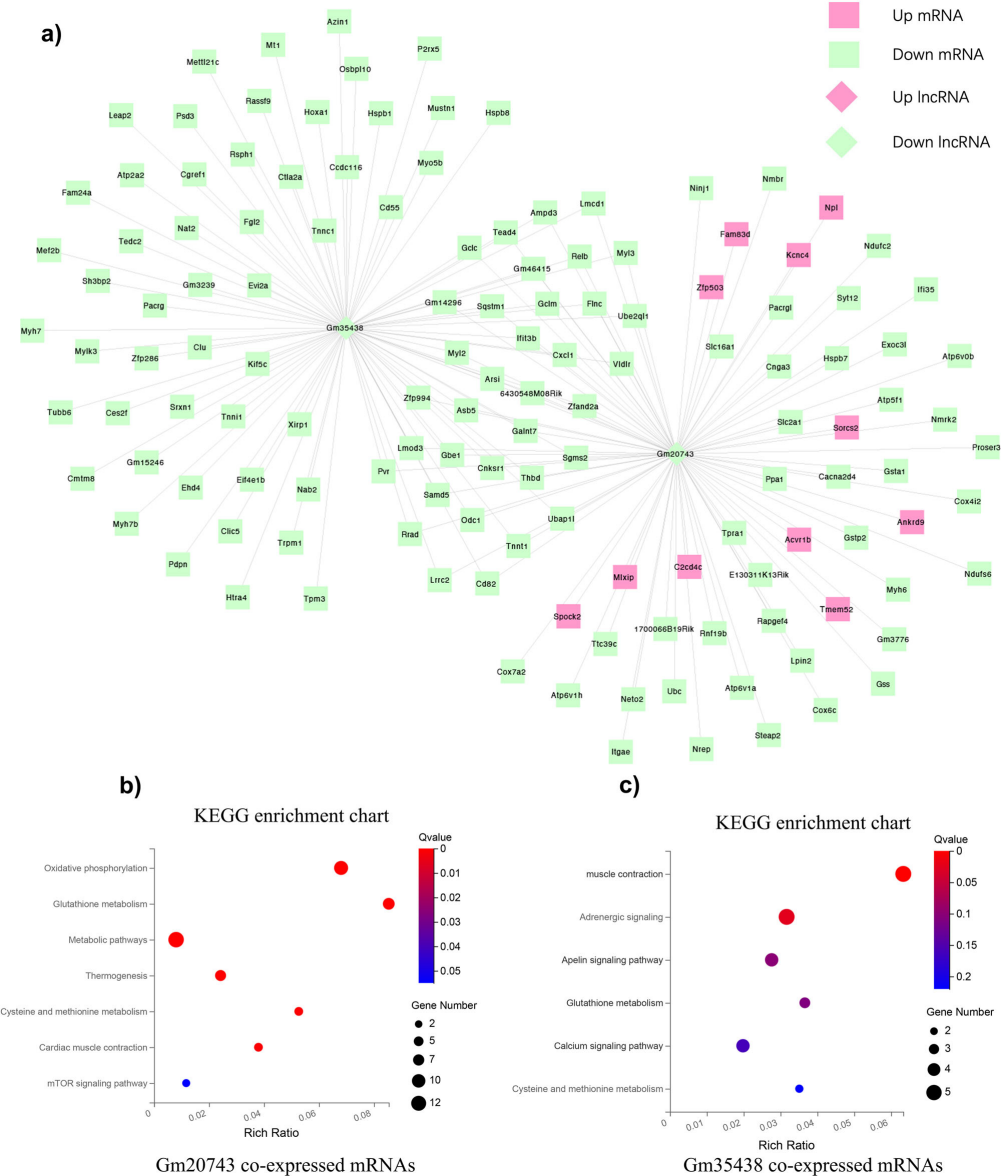
Co-expression network analysis and functional prediction of lncRNA Gm20743 and Gm35438. **a** The lncRNA Gm35438 and Gm20743 and 137 mRNAs were constructed in a co‐expression network with a PCC > 0.95. The red color represents upregulated genes, and the green color indicates down-regulation expression. Moreover, the top significant pathway terms of dysregulated genes associated with Gm35438 and Gm20743were displayed according to KEGG analysis (**b**, **c**), respectively. Note: PCC: Pearson correlation coefficient.

**Fig. 8:**
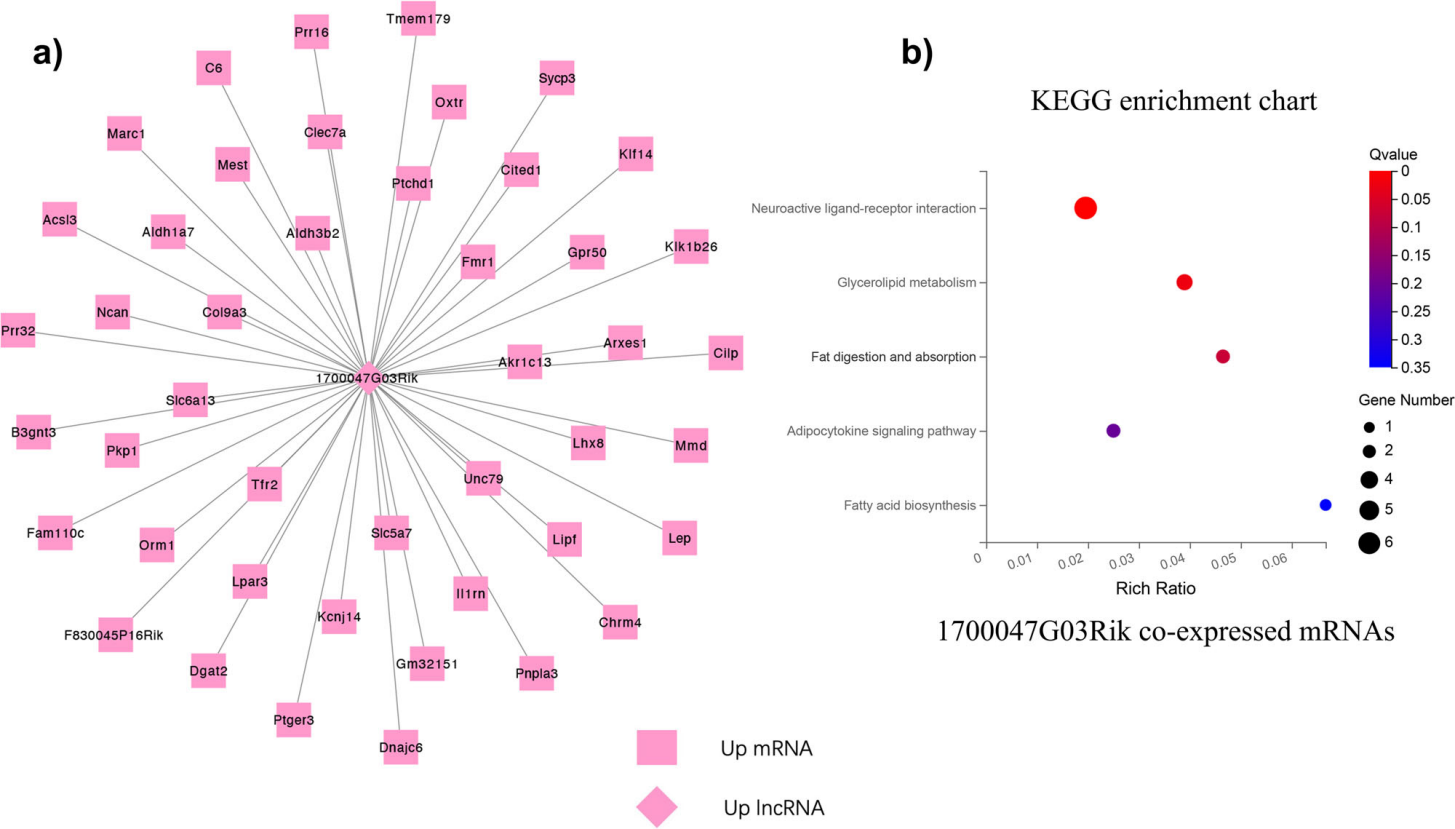
Co-expression network analysis and functional prediction of lncRNA 1700047G03Rik. **a** The lncRNA 1700047G03Rik and 45 mRNAs were constructed in a co‐expression network with a PCC > 0.95. **b** The top significant pathway terms of dysregulated genes associated with 1700047G03Rik were displayed according to KEGG analysis. Note: PCC: Pearson correlation coefficient.

Based on the co-expression relationship of lncRNA-mRNA, we generated diabetes-induced sarcopenia‐specific lncRNA‐mRNA co-expression networks. Through using correlation analysis of the expression, 24 lncRNA (log2 FC > 1, q-value < 0.05 and expression >2) and 860 mRNAs (Pearson correlation coefficient, PCC > 0.95, *p*-value < 0.05) were used to construct a highly correlated co-expression network. Several core dif-lncRNAs in the co-expression network were validated by RT-qPCR in *db/db* and *db/m* mice. The results showed that *1700047G03Rik* and *Gm31814* were upregulated, while *Gm20743, Gm35438, Gm36131*, and *A330074k22rik* were downregulated in GAS of the *db/db* group compared with *db/m* mice (Fig. 4a–f). Accumulated evidence showed that the elevated serum PA level in T2DM is involved in the development of insulin resistance and implicated in skeletal muscle inflammation, oxidative stress, and mitochondrial dysfunction by causing lipotoxicity, which leads to skeletal muscle loss^21,22^. In our results, PA-treated C2C12 myotubes decreased the diameter of and inhibited the differentiation level of myotubes using fusion index analysis (Fig. 5a, b). *Atrogin-1, MuRF-1*, and *Mstn*, which serve as muscle atrophy markers, were higher relative to NC (Fig. 5c–e). These results suggested that PA efficiently induced muscle atrophy in C2C12 myotubes. The expression of these six dif-lncRNAs was further confirmed in a PA-induced muscle atrophy C2C12 cell model by RT-qPCR (Fig. 6a–f). The results found that the expression of most lncRNAs was consistent with animal models and RNA-seq data except for *Gm38141* (Fig. 6e), which may be due to the differences in tissue and cellular components. Overall, the RT-qPCR validation of dysregulated lncRNAs of co-expression network in diabetes-associated sarcopenia mouse models and PA-treated C2C12 cells provide us with further indications to characterize their functions in the development of diabetes-associated sarcopenia. In addition, we found that 3 core dif-lncRNAs named *Gm20743* (log2FC = −1.57, *q*-value < 0.001), *Gm35438* (log2FC = −1.90, *q*-value < 0.001) and *1700047G03Rik* (log2FC = 6.34, *q*-value < 0.001) had the highest number of interactions in the co-expression network and may contribute to the development of diabetes-related sarcopenia (Figs. 7a and  8a).

### The potential function of candidate lncRNAs

We performed functional prediction of the co-expressed dif-mRNAs highly correlated with the three candidate lncRNAs by KEGG analysis, respectively. Functional analysis revealed that *Gm20743* may be involved in mitochondrial functions like oxidative phosphorylation and glutathione pathway (Fig. 7b). Through RT-qPCR verification, the key genes (like *ATP1B1, Gclc, Gclm*, and *GPX4*) in the co-expression network which contributed to oxidative phosphorylation and glutathione pathway were downregulated in vivo (Fig. 4g–j) and in vitro (Fig. 6g–j) consistent with the RNA-seq results. These results suggested that *Gm20743* may be involved in the regulation of mitochondrial function and redox homeostasis in skeletal muscle. KEGG analysis found that the dif-mRNAs co-expressed with *Gm35438* are mainly enriched in muscle contraction and adrenergic signaling pathways (Fig. 7c). The co-expressed contractile proteins *Tnnc1* and *TPM3* in these differentially enriched pathways were downregulated in *db/db* mice (Fig. 4k, l) and PA-treated C2C12 cells (Fig. 6k, l) compared to their control groups, and these results suggested that *Gm35438* may be related to skeletal muscle contraction and strength. Through co-expression network and bioinformatic analysis, we found the dif-mRNAs co-expressed with *1700047G03Rik* (the top upregulated lncRNA in Supplementary Table 10) were mainly enriched in the neuroactive ligand-receptor interaction and glycolipid metabolism pathway (Fig. 8b). The RT-qPCR validation found that co-expressed key catalytic enzymes *ACSL3* and *DGAT2* in the glycolipid metabolism pathway were upregulated in both animal (Fig. 4m, n) and cell models of diabetic sarcopenia (Fig. 6m, n). It is suggested that *1700047G03Rik* may be involved in the intramuscular lipid deposition of diabetes-related sarcopenia.

### The role of *Gm20743* in the development of diabetic sarcopenia

In this study, we specifically focused on the role of lncRNA *Gm20743* on mitochondrial function and redox homeostasis in skeletal muscle cells. The key reasons are (1) First, based on the criteria |log2 FC | > 0.585, FDR < 0.05, and expression >2, we ranked lncRNAs in terms of FDR and found that lncRNA *Gm20743* ranked in the top 20 differentially expressed lncRNAs. (2) Secondly, through a systematic literature review, many studies found that mitochondrial dysfunction and oxidative stress play an important role in sarcopenia; (3) Moreover, our previous study found that insulin resistance, mitochondrial dysfunction, and oxidative stress were involved in the development of muscle atrophy in PA-treated C2C12 cells^23,24^; (4) In addition, through functional prediction of dif-mRNAs and lncRNA-mRNA co-expression network. These results showed that the down-regulated mitochondrial oxidative phosphorylation, citrate cycle, and glutathione metabolism were the most significantly enriched pathways based on GSEA (Supplementary Figure 1c, e, f). The KEGG analysis also revealed that the genes highly correlated with *Gm20743* were mainly enriched in down-regulated oxidative phosphorylation and glutathione metabolism pathways (Fig. 7b). The above findings suggested that *Gm20743* may play an important role in regulating mitochondrial dysfunction and oxidative stress in diabetic sarcopenia.

**Fig. 9:**
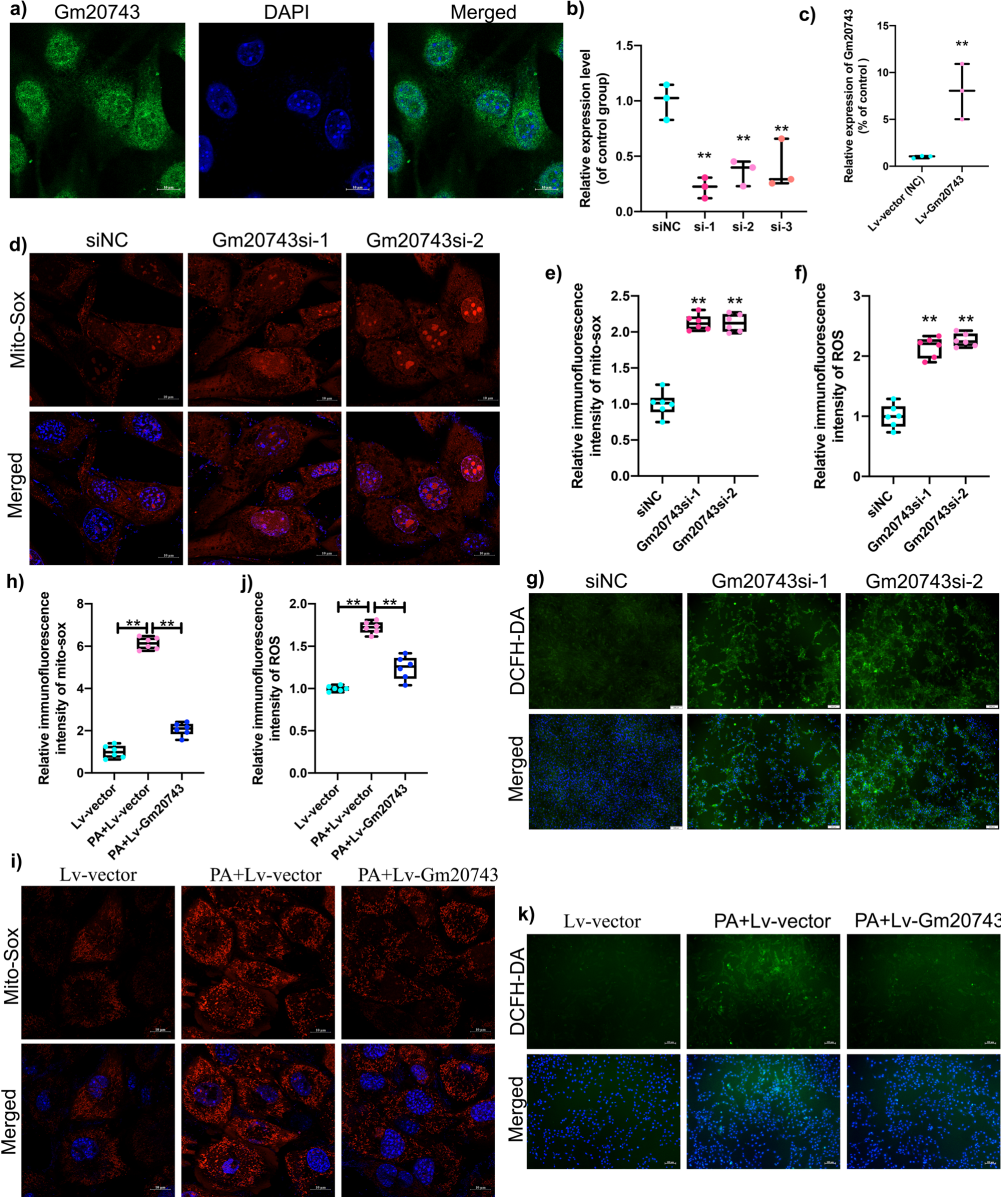
LncRNA Gm20743 regulates mitochondrial function and redox homeostasis in C2C12 cells. **a** Fluorescence in suit hybridization was used to observe the distribution of Gm20743 in C2C12 cells. The expression of Gm20743 in C2C12 cells transfected with Gm20743-siRNA (**b**) and Lv-Gm20743 (**c**) was determined by RT-qPCR. Mito-Sox staining was applied to detect mitochondrial ROS levels in C2C12 cells with Gm20743-siRNA (**d**, **e**), scale bar = 10 μm. DCFH-DA staining was performed to evaluate intracellular ROS levels in C2C12 cells with Gm20743-siRNA (**f**, **g**), scale bar = 100 μm. Mito-Sox staining was applied to detect mitochondrial ROS levels in C2C12 cells with Lv-Gm20743 (**h**, **i**), scale bar = 10 μm. DCFH-DA staining was performed to evaluate intracellular ROS levels in C2C12 cells with Lv-Gm20743 (**j**, **k**), scale bar = 100 μm. Data were expressed as mean ± S.D. Compared with the siNC or Lv-vector (NC) group, *P < 0.05 **P < 0.01. Note: si-1: Gm20743siRNA-1; si-2: Gm20743siRNA-2; si-3: Gm20743siRNA-3; DCFH-DA: 2’, 7’-dichlorofluorescein-diacetate; ROS: reactive oxygen species.

**Fig. 10:**
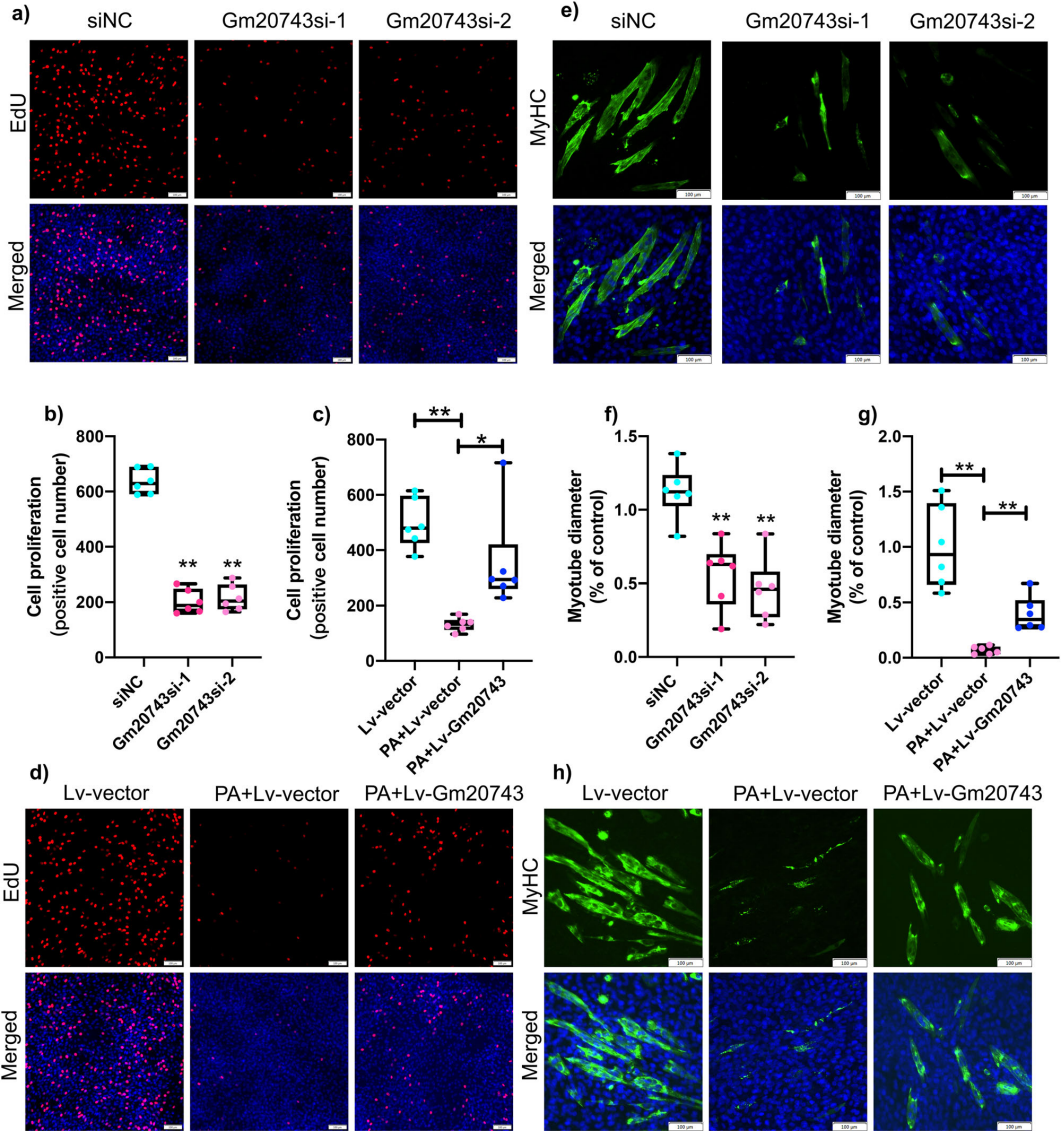
LncRNA Gm20743 regulates cell proliferation and myotube differentiation in C2C12 cells. EdU staining was measured to evaluate cell proliferation in C2C12 cells with Gm20743-siRNA (**a**, **b**) and Lv-Gm20743 (**c**, **d**), scale bar = 100 μm. Immunofluorescence staining of MyHC was determined to test myotube differentiation with Gm20743-siRNA (**e**, **f**) and Lv-Gm20743 (**g**, **h**), scale bar = 100 μm. Data were expressed as mean ± S.D. Compared with the siNC or Lv-vector (NC) group, *P < 0.05 **P < 0.01. Note: si-1: Gm20743siRNA-1; si-2: Gm20743siRNA-2; si-3: Gm20743siRNA-3; EdU: 5-ethynyl-2’-deoxyuridine; MyHC: myosin heavy chain.

To verify the role of *Gm20743* in diabetes-related sarcopenia, through fluorescence in suit hybridization (FISH), our results found that the distribution of *Gm20743* was found in both the cytoplasm and nucleus of skeletal muscle C2C12 cells (Fig. 9a). *Gm20743* was knocked down in C2C12 cells by siRNA transfection. The expression of *Gm20743* was significantly downregulated by *Gm20743*si-1, *Gm20743*si-2, and *Gm20743*si-3 in C2C12 cells (Fig. 9b), and *Gm20743* was overexpressed in C2C12 cells by lentiviral transfection and the expression of Lv-*Gm20743* was verified by RT-qPCR (Fig. 9c). After knocking down *Gm20743*, we found that the extent of knockdown of *Gm20743*si-1, and *Gm20743*si-2 was lower in C2C12 cells. To avoid off-target situations, we used *Gm20743*si-1 and *Gm20743*si-2 to knock down *Gm20743* in C2C12 cells, respectively. Through the detection of Mito-Sox and 2’, 7’-dichlorofluorescein-diacetate (DCFH-DA), our results found that knockdown *Gm20743* increased mitochondrial reactive oxygen species (ROS) (Fig. 9d, e) and intracellular ROS (Fig. 9f, g) in C2C12 cells with *Gm20743*si-1 and *Gm20743*si-2, while overexpression of *Gm20743* significantly reduced mitochondrial ROS (Fig. 9h, i) and intracellular ROS (Fig. 9j, k) in PA-induced C2C12 cells. These results suggest that *Gm20743* can regulate mitochondrial function and redox homeostasis in C2C12 cells. Previous studies found that mitochondrial dysfunction and oxidative stress prevent the proliferation and differentiation in skeletal muscle myoblasts^25–27^. To further assess whether *Gm20743* is involved in the cell proliferation and myotube differentiation in skeletal muscle myoblasts, the detection of ethynyl-2′-deoxyuridine (EdU) and myosin heavy chain (MyHC) staining was determined. In our results, we found that knockdown *Gm20743* decreased the number of EdU-positive cells in C2C12 myoblasts compared to the siNC group (Fig. 10a, b), and overexpressed *Gm20743* increased the number of EdU-positive cells in PA-induced C2C12 myoblasts (Fig. 10c, d), suggesting *Gm20743* improves cell proliferation in C2C12 cells. In addition, we observed that knockdown *Gm20743* significantly decreased the number and diameter of myotube in C2C12 cells compared to the siNC group (Fig. 10e, f), while overexpressed *Gm20743* significantly increased the number and diameter of myotube in PA-induced C2C12 myoblasts (Fig. 10g, h), compared to Lv-vector control. Together, these results indicate that *Gm20743* may promote cell proliferation and myotube differentiation by preventing mitochondrial dysfunction and oxidative stress in skeletal muscle cells.

## Discussion

Accumulating evidence illustrated that emerging lncRNAs were involved in skeletal muscle growth and regeneration^28–34^. However, the regulatory mechanism of lncRNAs in sarcopenia during diabetes pathogenesis remains unclear. Our study shows that thousands of differential gene expressions were found in *db/db* mice by whole transcriptome RNA-seq. Our results indicated that down-regulated “oxidative phosphorylation”, “metabolic pathways”, “muscle contraction” pathways and up-regulated “neuroactive ligand-receptor interaction”, and “glucolipid metabolism” pathways were significantly enriched in db/db mice according to functional enrichment analysis. We have revealed three core significant candidate lncRNAs *Gm20743, Gm35438, 1700047G03Rik*, and their potential function based on co-expression network and functional prediction. The candidate lncRNAs and their closely correlated gene expression were validated by RT-qPCR in *db/db* mice and PA-treated C2C12 cells. In this study, we examined the specific role of lncRNA *Gm20743* in skeletal muscle cells, and our results have shown that the level of mitochondrial and intracellular ROS in PA-treated C2C12 cells was reduced by overexpressed *Gm20743* and indicated that overexpressed *Gm20743* could prevent mitochondrial dysfunction and oxidative stress in C2C12 cells. The results also provided evidence that overexpressed *Gm20743* could promote cell proliferation and myotube differentiation in PA-induced C2C12 cells. All these data identify already known interactions and novel changes in the lncRNAs regulation in diabetes-related sarcopenia and an emphasis on the specific function of novel lncRNA *Gm20743* in the development of diabetes-related sarcopenia.

According to lncRNA-mRNA co-expression networks, we found three candidate lncRNAs *Gm20743, Gm35438*, and *1700047G03Rik* that may have vital regulatory roles in diabetes-related sarcopenia. Surprisingly, down-regulated lncRNA *Gm20743* is a key lncRNA with 86 co-expressed differential expression of genes (DEGs) in the co-expression network, and these highly correlated DEGs were mainly enriched in the down-regulated oxidative phosphorylation and glutathione pathway. Mitochondrial oxidative phosphorylation converts the energy released by nutrient oxidation into ATP. Studies indicated defective mitochondrial oxidative phosphorylation and ROS removal systems lead to ROS imbalance and oxidative stress, which in turn damages mitochondrial function in skeletal muscle^35,36^. Numerous studies also have reported that the complication of obesity and diabetes induces mitochondrial dysfunction with the activation of oxidative stress, ultimately leading to decreased muscle mass and strength^37–39^. Our results found that the down-regulated oxidative phosphorylation pathway was the most significantly enriched in *db/db* mice based on GSEA (Supplementary Figure 1c). In addition, the altered mitochondrial morphology was found in GAS tissue of *db/db* mice compared to the control group by TEM (Fig. 1m, n). These results were consistent with the performance of mitochondrial dysfunction in diabetic skeletal muscle. A recent study had described that the glutathione pathway plays a positively substantial role in removing ROS and skeletal muscle mass by modulating redox homeostasis^40–43^ which is consistent with our findings with the down-regulated glutathione pathway in *db/db* mice. Moreover, the downregulated expression of highly co-expressed genes (such as *ATP1b1, Gclc, Gclm*, and *GPX4*) closely related with *Gm20743* was consistent with downregulated oxidative phosphorylation and glutathione pathway^44,45^. Therefore, we more focused on the specific function of lncRNA *Gm20743* on mitochondrial function and oxidative stress in this study. Through in vitro experiments, Lv-*Gm20743* C2C12 cells exhibited a lower mitochondrial and intracellular ROS accumulation in PA-treated atrophic muscle cells. The imbalance in the enzymatic activity of mitochondrial complexes, mitochondrial complexes I and III generate ROS production^46,47^. Although our study did not provide sufficient direct evidence, it has been proposed that high levels of mitochondrial ROS lead to downregulation of mitochondrial membrane potential and further decreased mitochondrial oxidative phosphorylation activity, ultimately leading to mitochondrial dysfunction^36^. These findings suggest that overexpressed *Gm20743* ameliorate mitochondrial function and oxidative stress via decreasing the production of mitochondrial and intracellular ROS. Thus, these findings strongly suggest that overexpressed *Gm20743* has a very important role in the regulation of mitochondrial function and redox homeostasis via a lower level of mitochondrial and intracellular ROS in PA-treated C2C12 cells. Moreover, the results demonstrate that overexpression of *Gm20743* improves cell proliferation and myotube differentiation in PA-treated skeletal muscle cells. Collectively, it is important to highlight the fact that *GM20743* may be involved in promoting mitochondrial function and redox homeostasis to regulate cell proliferation and myotube differentiation in skeletal muscle cells. However, the function of *Gm20743* has not been observed in skeletal muscle of any disease models in public databases such as GEO profiles and GTEx. To our knowledge, this is the first time we report the specific function of *Gm20743* in mouse skeletal muscle cells. In addition, through gene alignment in the public human database, we found that some sequences on human chromosome 1 were highly homologous to the sequences of lncRNA *Gm20743* on mouse chromosome 1, and the proportion of homologous genes reached 70-80%. Therefore, this study on the role of lncRNA *Gm20743* in diabetes-related sarcopenia makes it very meaningful to further explore the human gene homologous to *Gm20743* as a target or biomarker for clinical diagnosis and therapy in the future.

Our results also revealed the potential functions of the other two candidate *lncRNAs Gm35438* and *1700047G03Rik* according to the co-expression networks. The KEGG enrichment analysis revealed that 86 DEGs co-expressed with *Gm35438* were mainly enriched in muscle contraction and adrenergic pathways. Exactly as the muscle contraction pathway, the adrenergic signaling pathway is also considered to regulate skeletal muscle contraction and muscle mass through excitation-contraction coupling^48^. The key contractile proteins troponin C1 (Tnnc1) and tropomyosin3 (TPM3) which are involved in both muscle contraction and adrenergic signaling pathways were down-regulated in the *Gm35438*-mRNA co-expression network. Tnnc1 is one of the TnC isoforms encoded by homologous genes in slow skeletal and cardiac muscle^49^. Tnnc1 has also been recognized as a key biological marker for assessing muscle fiber type and quality^50^. TPM3 is a slow-twitch muscle fiber tropomyosin involved in skeletal muscle contraction^51^. Studies have found that maximal contractile force in skeletal muscle is decreased in patients with TPM3 mutations^52,53^. Compared to their controls, downregulated expression of Tnnc1 and TPM3 were validated in *db/db* mice (Fig. 4k, l) and PA-treated C2C12 cells (Fig. 6k, l) which is consistent with the RNA-seq data. Thus, we speculate that *Gm35438* is likely involved in the regulation of skeletal muscle contraction and needs further explorations in the future. Our results showed that 45 co-expressed DEGs were closely correlated to core lncRNA *1700047G03Rik*, which were mainly enriched in the up-regulated neuroactive ligand-receptor interaction and glycolipid metabolism pathways based on KEGG analysis. The results showed that up-regulated neuroactive ligand-receptor interaction pathway is the most differentially enriched pathway in db/db mice based on GSEA (Supplementary Fig. 1a). Although a clear correlation of neuroactive ligand-receptor interactions in skeletal muscle atrophy or sarcopenia has not yet been reported, it may be a new mechanism of diabetes-related sarcopenia. Recent studies have demonstrated that sarcopenia is accompanied by accumulated intramuscular fat (IMAT) infiltration^54^. In skeletal muscle, IMAT is a common feature in several myopathies and is related to muscular dysfunction and insulin resistance^55–57^. *ACSL3* and *DGAT2* are localized on the endoplasmic reticulum and act as a key catalytic enzyme to promote the synthesis of lipids and triglycerides^58,59^. A study found that increased lipid metabolism pathways such as the glycolipid pathway may contribute to insulin resistance and muscular dysfunction, leading to skeletal muscle loss and low muscle strength^60^. Consistent with the RNA-seq results, the up-regulated expressions of *ACSL3* and *DGAT2* which are involved in the co-expression network with core *1700047G03Rik* were confirmed by RT-qPCR in *db/db* mice (Fig. 4a, m, n) and PA-treated C2C12 cells (Fig. 6 a, m, n) compared with their controls. These results supported that *1700047G03Rik* related to the function of *ACSL3* and *DGAT2* which are involved in lipid synthesis may play vital roles in diabetes-related sarcopenia. Therefore, we speculate that *1700047G03Rik* may regulate lipid metabolism to change fatty acid content or composition in skeletal muscle, ultimately causing skeletal muscular dysfunction, atrophy, and weakness, and further verification will be needed in the future.

Under physiological conditions, skeletal muscle mass and muscle strength reach their peak in the 20 s to 40 s of age in human life^61^. The positive prevention of age- or disease-related sarcopenia should begin in young and midlife and continue throughout life to avoid irreversible severe adverse outcomes in the late stage. Therefore, we selected relatively young *db/db* mice for observation in this study, with the hope to find novel therapeutic targets and prevention strategies for diabetes/obesity-related sarcopenia at an early stage. The leptin-deficient *db/db* mouse model used in our study has previously been shown to be affected by the metabolic disorders of obesity and T2DM, including elevated blood glucose and abnormal insulin secretion^62,63^. In our data, the DEXA test has found that the lean mass of 15-week-old *db/db* mice is significantly lower than that of control *db/m*, accompanied by a decrease in forelimb grip strength, although *db/db* mice are severely obese and overweight. These results strongly demonstrated that muscle atrophy is evident in *db/db* mice of the T2DM model. Thus, the *db/db* mice are a suitable and reliable model for obesity and diabetes-induced sarcopenia. Our data showed that reduced skeletal muscle mass and strength, decreased CSA, increased body fat and a high level of triacylglycerols were found in *db/db* mice, and a recently published study by Pandeya confirms our findings in this study^64^. Based on the available literature, several RNA-seq research on skeletal muscle insulin resistance were performed in vivo and in vitro, and these studies are mainly aimed at detecting the novel changes of lncRNAs and their potential function in insulin-resistant skeletal muscle^65,66^. Compared with these studies, the strengths of our investigation include (1) we predicted the regulation of three key candidate lncRNAs (*Gm20743, Gm35438, 1700047G03Rik*) with potential significant pathways in diabetes-related sarcopenia. (2) our study focused on the specific function of novel lncRNA *Gm20743* in the development of diabetes-related sarcopenia. Compared to these published RNA-seq studies, we identified partial inconsistencies in the dif-lncRNAs, dif-mRNAs, and significantly enriched pathways in our study, which may be related to differences in muscle tissue composition, PA concentration, time of intervention, and the stage or development of insulin resistance, diabetes, and muscle atrophy.

However, this study has certain limitations. Our study underscores the ability of whole transcriptome RNA-seq analysis to provide specific information in diabetes-induced sarcopenia. The *db/db* mouse model is a well-known model of T2DM with the phenotype of severe obesity, this model is considered as obesity and T2DM are both linked to sarcopenia. In this study,15-week-old *db/db* mice exhibit a sarcopenic status with T2DM and obesity, the skeletal muscle of *db/db* mice is compromised and demonstrates a T2DM/obesogenic muscle dysregulation in young mice with a sarcopenic phenotype. However, *db/db* mice model cannot present aging skeletal muscle status. Although the sequencing data is derived from *db/db* mice, the mouse pathological phenotype already closely mimics human obesity and T2DM-induced sarcopenia. The state of T2DM can partially present the pathophysiological process of human diabetes-induced sarcopenia during midlife. Despite the small sample size, given the sequencing depth obtained per sample, we believe that our results could provide complex and comprehensive information and insight into differential gene expression profiles in overt obese and diabetes db/db mice. Future studies with the consideration of human skeletal muscle samples and increased sample size would allow further analysis to determine gene signatures that correspond to differential responses to diabetic sarcopenia in different disease stages.

## Materials and Methods

### Animals

Eight-week-old male *db/db* mice (C57BLKS- Lepr^db/db^ diabetic mice) (*n* = 10) and *db/m* male wild-type littermates (C57BLKS- Lepr^db/m^ mice) (*n* = 10) were purchased from SPF Biotechnology Company (Beijing, China). The *db/db* mice model is a well-known model of T2DM with heavily obese. *Db/m* mice were considered as the normal control group. All mice were fed with a standard chow diet (18 kcal% fat, 24 kcal% protein, 58 kcal% carbohydrates; Research Diets, Inc) for seven weeks. 15-week-old *db/db* mice model exhibit a sarcopenic status with T2DM and obesity during young and midlife. Bodyweight and blood glucose were measured. Grip strength was measured by using an electronic grip strength metre (Cat. 47200, Ugo Basil) as described previously^23^. The grip index is reported as grip strength divided by body weight. Body composition was measured as described previously^23^ by DEXA (Hologic Discovery A, Hologic Inc) (*n* **=** 5). After fasting for 6 h, mice were anaesthetized and sacrificed with 2% pentobarbital sodium solution at fifteen-week of age. Blood was harvested, and serum biochemical indexes were determined by an automatic biochemistry analyser (*n* **=** 5). GAS was harvested and stored in liquid nitrogen for RNA‐seq and molecular biological examinations. Approval for this research was obtained by the Ethics Committee of Chongqing Medical University and the First Affiliated hospital of Chongqing Medical university (Chongqing, China), and all procedures complied with the guidelines and principles for the care and use of laboratory animals approved by Chongqing Medical University.

### Histological analysis

GAS was fixed in 4% paraformaldehyde for 24 h, dehydrated in gradient alcohol, and then embedded in paraffin. The samples were cut into 6-μm-thick sections. GAS sections were stained with H&E and Masson trichrome, as described previously^67^. Frozen sections were stained with Oil-red O^23^. The average CSA, fibrosis, and Oil-red O-positive area of GAS myofibres were measured and analyzed by ImageJ software (NIH, USA).

### TEM

GAS was cut into 1 mm^3^ cube and fixed rapidly. After dehydration, infiltration, embedding, and polymerization, samples were processed for TEM analysis as described previously^23^. The images were taken at ×20,000 and ×50,000 magnification by a transmission electron microscope (HT7700, HITACHI).

### RNA isolation and RNA-seq

Total RNA of GAS (medial and lateral portions) was extracted from each mouse (each group *n* = 3) using TRIzol (Invitrogen) according to the instructions. The integrity and purity of total RNA were measured by using a NanoDrop and Agilent 2100 bioanalyzer (Thermo Fisher Scientific). RNA samples with RIN ≥ 8 and 28 S/18 S ≥ 1.5 were sequenced. mRNA was purified by using a Dynabeads mRNA Purification Kit (Invitrogen). The products were amplified using PCR to construct the final cDNA library. The qualified cDNA library was constructed and sequenced using the paired-end mode on the BGISEQ-500/MGISEQ-2000 System of the BGI–Tech Bioinformatics Institute (Shenzhen, China).

### Analysis of RNA-seq data

All profiling analyses for RNA‐seq were processed with the assistance of BGI. The raw sequencing data were mapped to the reference genome by using the HISAT2 programme. The raw data were aligned to the database built by BGI, and the expression level of genes was calculated by using the RSEM programme. DESeq2 was used to analyse the DEGs with the following criteria: |log_2_ FC | > 0.585, and *q*-value or FDR < 0.05. The heatmap was generated by using the pheatmap (v1.0.8) programme according to the gene expression in each sample.

### Functional enrichment analysis of DEGs

GO enrichment analysis was performed to construct the three main functions of differentially expressed mRNAs (dif-mRNAs) (*q*-value < 0.05). KEGG pathway enrichment analysis was conducted to obtain pathway sets containing dysregulated mRNAs (*q*-value < 0.05) in the molecular biological mechanism network. An enrichment score with a −log 10 (*q*-value) value denotes significance for the GO terms and KEGG pathways (*q*-value < 0.05). GSEA was performed to determine novel and potential biological functions with a predefined set of genes (*p*-value  < 0.05).

### Construction of co-expression networks

Co‐expression analysis of dif-mRNAs and dif-lncRNAs was based on the calculation of the PCC^68^. Dif-lncRNAs and dif-mRNAs (*q*-value < 0.05) with a PCC > 0.95 were selected to construct lncRNA-mRNA co‐expression networks. Visual networks were produced with Cytoscape software.

### Cell culture and lentiviral transfection

Mouse C2C12 myoblasts (ATCC, USA), skeletal muscle precursor cells with the potential for myotube differentiation and muscle regeneration, were cultured in Dulbecco’s modified Eagle’s medium (DMEM, Gibco, USA) containing 10% foetal bovine serum (Bioind, Israel), and 1% penicillin–streptomycin (Gibco, USA). All cells were incubated in 5% CO_2_ atmosphere at 37 °C. After the cells reached 70–95% confluence, the culture medium was switched to a differentiation medium with DMEM containing 2% horse serum (Bioind, Israel) to induce differentiation into myotubes. Myotubes appeared after 4–5 days of differentiation and were used for subsequent experiments. The *Gm20743*-siRNAs and Scrambled nontargeting siRNA (siNC) were transfected into C2C12 myoblasts in 6-well plates by using EndoFectin™‐MAX Transfection Reagent (GeneCopoeia, USA) according to the manufacturer’s protocol. The siRNAs *Gm20743*-siRNA1, *Gm20743*-siRNA2, and *Gm20743*-siRNA3 were purchased from RiboBio (Guangzhou, China). Scrambled nontargeting siRNA was used as a negative control provided by RiboBio (Guangzhou, China). The sequence of *Gm20743*-siRNAs was as shown: *Gm20743*-siRNA1: 5′‐GGACTATCAGATCATGTTT‐3′, *Gm20743*-siRNA2: 5′‐GCCAAGAATGCAGAACAAA‐3′, *Gm20743*-siRNA3: 5′‐CTTCCACATACTAGACAAA‐3′. The extent of *Gm20743* knockdown was measured and analyzed by RT‐qPCR. For stable overexpression, lentiviral vectors were used for gene delivery. The recombinant lentiviral vectors HBLV-m-*Gm20743*-Null-PURO (Lv-*Gm20743*), HBLV-ZsGreen-PURO (Lv-Green-vector), and HBLV-PURO (Lv-vector) were purchased from HanBio Technology Co., Ltd. (Shanghai, China). According to the transfection of the multiplicity of infection via Lv-Green-vector, the lentiviruses Lv-*Gm20743* and Lv-vector were each added separately into the culture medium of C2C12 myoblasts at an MOI of 10. Stably transfected cells were selected by the addition of 4 μg/ml puromycin (HanBio, Shanghai, China) until uninfected control cells were completely dead. PA was diluted with a differentiation medium that contained fatty acid-free bovine serum albumin (BSA; Sigma-Aldrich, USA). Consistent with the concentration of PA and intervention time of insulin resistance cell model in our previous study^23,24^, C2C12 cells were treated with 0.5 mM PA (Sigma–Aldrich, USA) for 24 h. The experiments are grouped into NC group, PA-treated group, Lv-vector group, PA + Lv-vector group, and PA + Lv-*Gm20743* group. All experiments were repeated three times.

### RT-qPCR analysis

cDNA was synthesized from total RNA of GAS (*n* = 5) by reverse transcription reactions with a PrimeScript RT Reagent Kit (Tiangen, China). RT‐qPCR was processed with SYBR Premix (Tiangen, China) by using a CFX Real‐Time PCR Detection System (Bio‐Rad, CA). Primers are presented in Supplementary Table 11. The equation 2^-ΔΔCt^ was used to calculate the relative fold changes of RNA expression. ACTB and GAPDH were performed as reference genes. The mean values of the control group were set to 1. All experiments were repeated at least three times.

### FISH

After seeding, cells were incubated with 4% paraformaldehyde for 15 min and then permeated with 0.3% Triton for 15 min. Prehybridized with specific solutions in a FISH kit (RiboBio, China), and hybridized with FAM-labeled lncRNA *Gm20743* probe (5 μg/mL) overnight at 37 °C (Genepharma, China). Subsequently, cells were washed three times with 4× SSC detergent containing 0.01% Tween-20 at 42 °C. After washing with 2× SSC and 1×SSC detergent at 42 °C, cells were stained with DAPI. Cells with FAM-labeled probes were detected by using confocal laser scanning fluorescence microscopy (ZEISS, LSM800, Germany).

### Immunofluorescence

Immunofluorescence staining was performed as described previously^23^. After incubation with anti‐MyHC antibody (1:200, Sigma–Aldrich, USA) overnight at 4 °C, C2C12 myotubes were incubated with Alexa Fluor 488‐conjugated goat anti‐mouse IgG antibody (1:400, ZSGB‐BIO, China) for 2 h. The nuclei were stained with DAPI (Beyotime, China). Myotubes with at least two nuclei were considered MyHC-positive myotubes (>2 nuclei). Images were observed by confocal laser scanning fluorescence microscopy (ZEISS, LSM800, Germany). The diameter of the myotubes was measured by ImageJ software (NIH, USA).

### ROS and Mito-Sox

The intracellular ROS in C2C12 cells was measured by using a ROS Assay Kit (Beyotime, China). 2’, DCFH-DA, which is the ROS assay kit principal component, is oxidized easily to fluorescent dichlorofluorescein by cellular ROS. Therefore, intracellular ROS levels were detected. The mitochondrial ROS in C2C12 cells were detected by a Mito-Sox Assay Kit (Invitrogen, USA). According to the manufacturer’s protocol, C2C12 cells were washed with serum-free medium 3 times and treated with DCFH-DA and Mito-Sox for 20 mins respectively. After washing, C2C12 cells were stained with Hoechst (Beyotime, China) for 5 mins and then intracellular ROS was observed by fluorescence microscopy (Olympus, Japan) and mitochondrial ROS was observed by confocal laser scanning fluorescence microscopy (ZEISS, LSM800, Germany). The relative fluorescence intensive was measured by ImageJ software (NIH, USA).

### Detection of EdU assay

After the intervention, C2C12 cells seeded in 6-well plates were washed 3 times. According to the manufacturer’s protocol, C2C12 cells were fixed and stained with an EdU Cell Proliferation Kit with Alexa Fluor 594 (Beyotime, China). The number of EdU-positive cells was observed by fluorescence microscopy (Olympus, Japan). The EdU-positive cell numbers were measured by ImageJ software (NIH, USA).

### Statistics and reproducibility

All data are presented as the mean ± standard deviation (S.D.). GraphPad Prism (San Diego, CA) was used to analyze the data. The comparisons of the two groups were detected by using an unpaired two-tailed Student’s t-test. Three or more groups were measured by one-way analysis of variance. Pearson’s correlation analysis was used to detect the correlation between two groups. The significance was regarded as *p*-values < 0.05. All experiments were repeated at least three times.

## Supplementary information

Supplementary Information

Description of Additional Supplementary Files

Supplementary Data 1

Supplementary Data 2

Supplementary Data 3

Supplementary Data 4

Supplementary Data 5

Reporting-summary

## Data Availability

The original RNA sequencing data of this manuscript was uploaded to the database of Sequence Read Archive (SRA) successfully, and the accession number is PRJNA778195. The source data for all figures in main text was in Supplementary Data 5.
