# Supplementary material for: The Whole-transcriptome Landscape of Diabetes-related Sarcopenia Reveals the Specific Function of Novel lncRNA Gm20743
Source: Commun Biol. 2022 Aug 1;5:774. doi: 10.1038/s42003-022-03728-8 (PMC9343400; doi:10.1038/s42003-022-03728-8)
Supplement: Supplementary file 1 — Supplementary Information [file 42003_2022_3728_MOESM1_ESM.pdf]

Supplementary Materials for

The Whole-transcriptome Landscape of Diabetes-related Sarcopenia Reveals the Specific Function of Novel lncRNA Gm20743

Jing Yu, Kim Loh, He-qin Yang, Meng-ran Du, Yong-xin Wu, Zhi-yin Liao, Ai Guo, Yun-fei Yang, Bo Chen, Yu-xing Zhao, Jin-liang Chen, Jing Zhou, Yue Sun, Qian Xiao*

* **Correspondence:** Qian Xiao. e-mail: xiaoqian1956@126.com; Tel.: +86 23 89011632.

Supplementary Figures

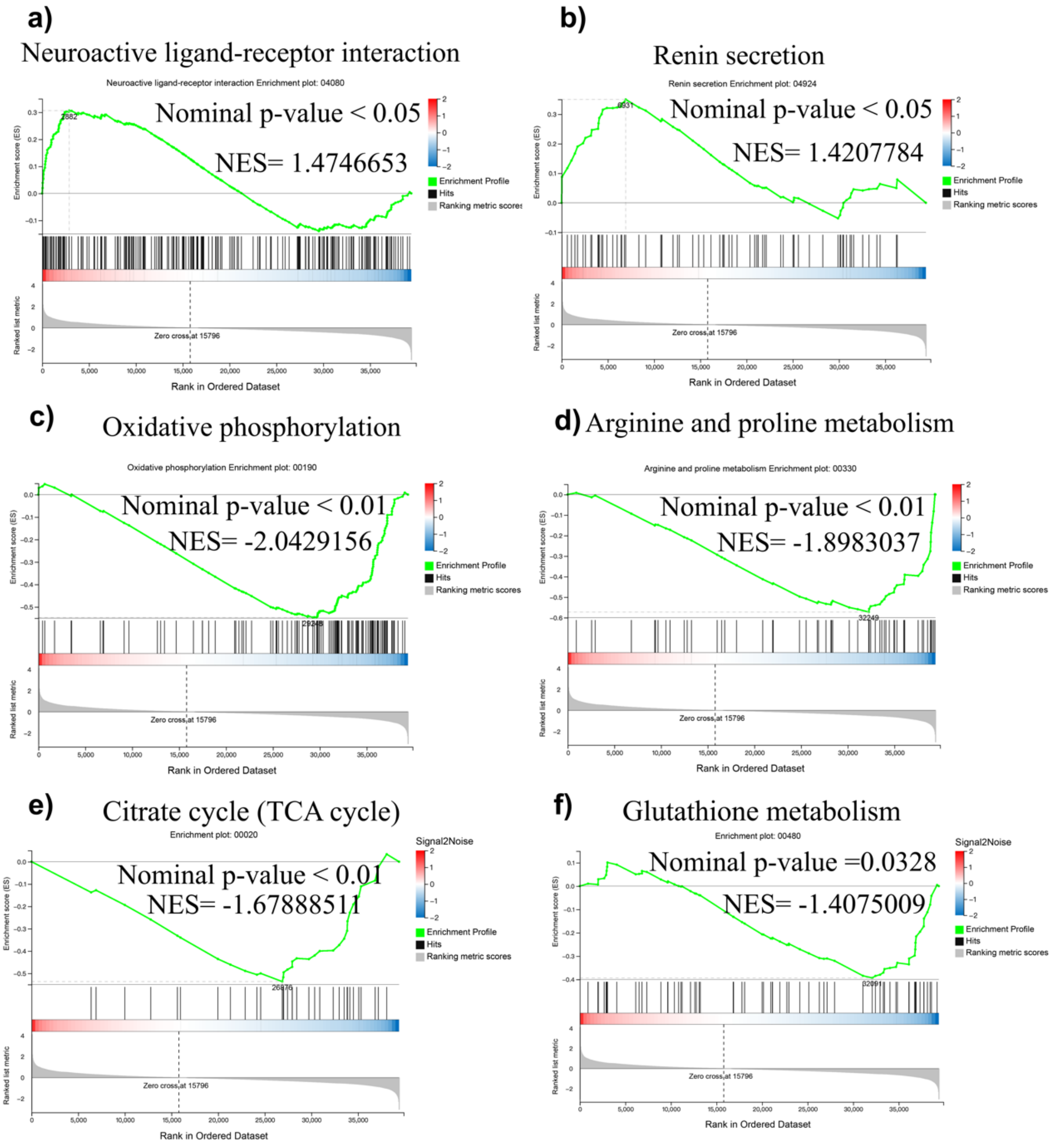

Supplementary Figure 1. Gene set enrichment analysis of DEGs. The top 2 dysregulated pathways of GSEA: **a)** neuroactive ligand-receptor interaction, **b)** renin secretion, **c)** oxidative phosphorylation and **d)** arginine proline metabolism in *db/db* vs. *db/m*. The pathway of **e)** citrate cycle and **f)** Glutathione metabolism was significantly enriched based on GSEA. The ES was exhibited by green curves for each gene shown by a black line, and are ranked all the differential expressions indicating the observed fold change in GAS from *db/db* vs. *db/m*. The NES and nominal p-value are displayed. *Note*: GSEA: Gene set enrichment analysis; ES: enrichment score; NES: normalized enrichment scores.

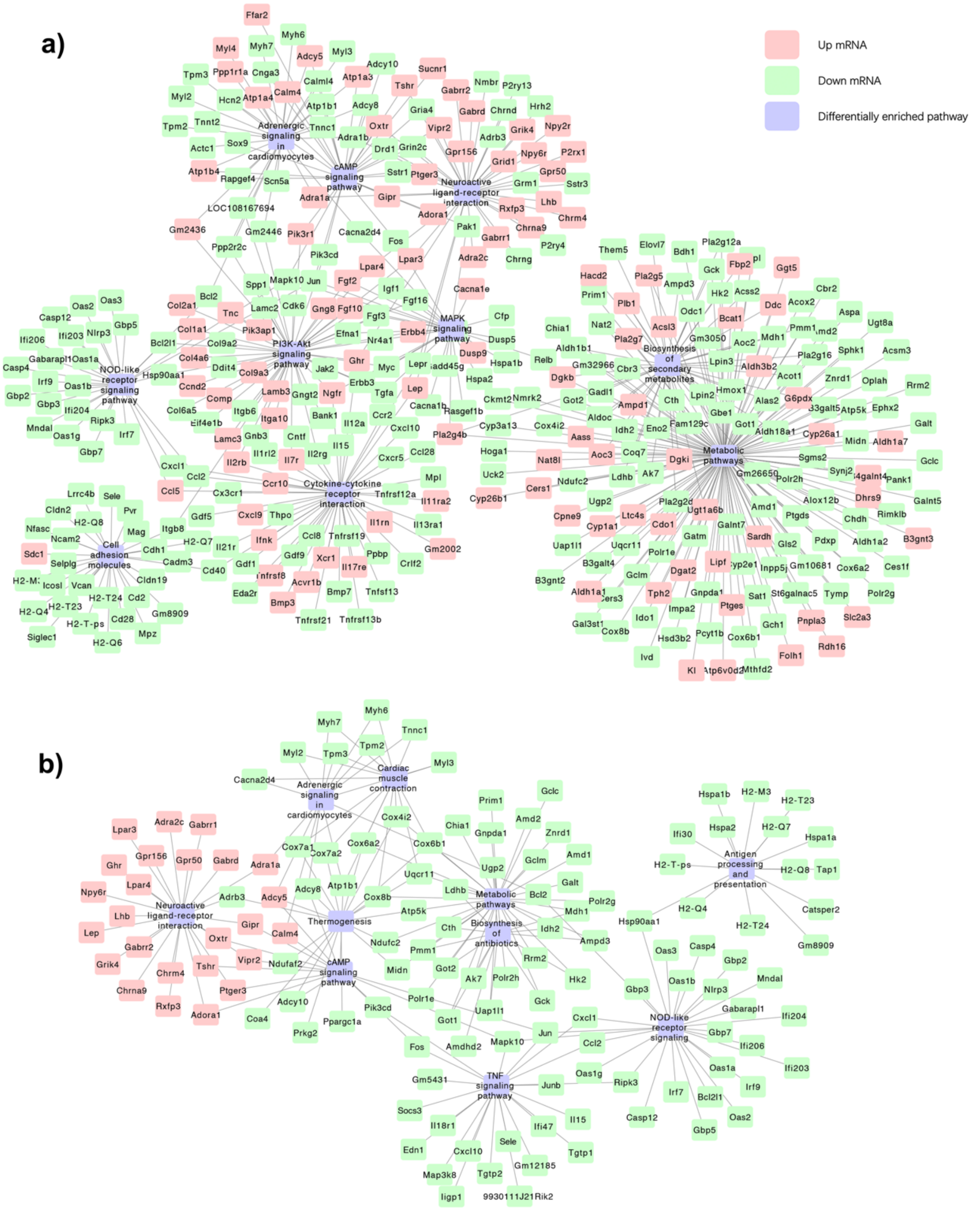

Supplementary Figure 2. Pathway-act-network in DEGs. a) The links and overlaps among differentially expressed mRNAs in significant pathway terms of KEGG and **b)** a visual pathway-act-network were constructed with DEGs which involved in10 significant pathways of GSEA.

Supplementary Tables

Supplementary Table 1. Serum biochemical parameters of the db/db vs. db/m (n=5)

Variable	db/db	db/m
**Total cholesterol (mM)**	4.06±0.47*	2.31±0.25
**Glucose (mM)**	33.73±7.79*	7.55±3.68
**Triglycerides (mM)**	1.31±0.11*	0.91±0.05
**HDL cholesterol (mM)**	2.88±0.27*	1.60±0.08
**LDL cholesterol (mM)**	0.60±0.07*	0.49±0.08

Note: HDL=high-density lipid; LDL= low-density lipid. Results are presented as mean ± standard deviation; * P ＜ 0.05.

Supplementary Table 2. Mapping statistics of RNA sequencing reads

Sample	dbdb1	dbdb2	dbdb3	dbm1	dbm2	dbm3
**Total Raw Reads (M)**	114.94	112.44	114.94	114.94	112.44	114.94
**Total Clean Reads (M)**	113.79	111.56	113.97	113.92	111.47	113.86
**Total Mapping (%)**	97.78	97.87	97.9	97.83	97.9	97.85
**Uniquely Mapping (%)**	90.97	90.38	90.62	89.81	88.88	89.93
**Clean Reads Ratio (%)**	98.99	99.22	99.16	99.11	99.13	99.06
**Total Clean Bases (Gb)**	11.38	11.16	11.4	11.39	11.15	11.39
**Clean Reads Q20 (%)**	98.44	98.22	98.25	98.41	98.38	98.4
**Clean Reads Q30 (%)**	95.31	94.66	94.7	95.22	95.16	95.19
**Clean Reads Ratio (%)**	98.99	99.22	99.16	99.11	99.13	99.06

Supplementary Table 3. Top 20 differential expressed mRNAs in GAS of db/db vs. db/m (FDR <0.05)

Gene ID	Gene Symbol	Log2 FC	*p*-value	FDR	style
70892	Ttll7	0.918823264	0	0	Up
234214	Sorbs2	0.776843541	0	0	Up
269016	Sh3rf2	1.696918892	0	0	Up
116847	Prelp	1.146502172	0	0	Up
53412	Ppp1r3c	1.068268306	0	0	Up
27226	Pla2g7	1.331476808	0	0	Up
18708	Pik3r1	1.051489968	0	0	Up
170768	Pfkfb3	0.708544781	0	0	Up
382384	Odf3l2	2.170350263	0	0	Up
18162	Npr3	2.277106351	0	0	Up
74843	Mss51	1.408634922	0	0	Up
208104	Mlxip	0.640963126	0	0	Up
320878	Mical2	0.664784644	0	0	Up
319476	Lrtm1	1.523466221	0	0	Up
16846	Lep	4.93941041	0	0	Up
21847	Klf10	0.986282824	0	0	Up
16515	Kcnj12	0.884200701	0	0	Up
15439	Hp	1.734063754	0	0	Up
102634778	Gm32280	1.245366508	0	0	Up
14600	Ghr	0.626991956	0	0	Up
22437	Xirp1	-1.278725375	0	0	Down
22359	Vldlr	-1.245196515	0	0	Down
215031	Vgll2	-1.137359022	0	0	Down
216558	Ugp2	-0.785232547	0	0	Down
22229	Ucp3	-1.105311339	0	0	Down
22190	Ubc	-0.666272023	0	0	Down
23999	Twf2	-1.053594947	0	0	Down
433766	Trim63	-1.426733009	0	0	Down
211770	Trib1	-1.547888405	0	0	Down
59069	Tpm3	-1.543479567	0	0	Down
22004	Tpm2	-1.032750542	0	0	Down
21955	Tnnt1	-2.122315015	0	0	Down
21952	Tnni1	-1.635229506	0	0	Down
21924	Tnnc1	-2.098732782	0	0	Down
319801	Tigar	-0.695886503	0	0	Down
20975	Synj2	-1.275341424	0	0	Down
76650	Srxn1	-1.531650003	0	0	Down
18412	Sqstm1	-0.603312489	0	0	Down
20411	Sorbs1	-0.599277985	0	0	Down
228608	Smox	-1.705544133	0	0	Down

Note： mRNAs: messenger RNAs; FC: fold change; FDR: false discovery rate.

Supplementary Table 4. GO analysis of up-regulated mRNAs in GAS of db/db vs. db/m mice. Top 10 significantly changed terms were shown.

GO_C Term ID	GO_C Term Desc	GO_C Term Level1	GO_C Term Level2	Term Candidate Gene Num	Total Candidate Gene Num	Term Gene Num	Total Gene Num	Rich Ratio	*p*-value	FDR
GO:0005576	extracellular region	cellular_component	extracellular region	122	598	1885	22482	0.06472149	1.19E-20	4.98E-18
GO:0062023	collagen-containing extracellular matrix	cellular_component	extracellular region part	36	598	304	22482	0.11842105	6.87E-14	1.44E-11
GO:0005615	extracellular space	cellular_component	extracellular region part	99	598	1724	22482	0.05742459	2.07E-13	2.89E-11
GO:0031012	extracellular matrix	cellular_component	extracellular region part	30	598	280	22482	0.10714286	1.07E-10	1.12E-08
GO:0016021	integral component of membrane	cellular_component	membrane	255	598	7116	22482	0.03583474	6.88E-09	5.75E-07
GO:0016020	membrane	cellular_component	membrane	296	598	8846	22482	0.03346145	2.15E-07	1.50E-05
GO:0005581	collagen trimer	cellular_component	protein-containing complex	12	598	92	22482	0.13043478	5.87E-06	3.50E-04
GO:0099055	integral component of postsynaptic membrane	cellular_component	membrane	11	598	84	22482	0.13095238	1.38E-05	7.20E-04
GO:0043025	neuronal cell body	cellular_component	cell part	35	598	655	22482	0.05343511	8.18E-05	0.0038014
GO:0005887	integral component of plasma membrane	cellular_component	membrane	56	598	1269	22482	0.04412924	1.46E-04	0.0060839
GO:0005201	extracellular matrix structural constituent	molecular_function	structural molecule activity	19	573	123	21691	0.15447154	5.83E-10	4.38E-07
GO:0005520	insulin-like growth factor binding	molecular_function	binding	7	573	24	21691	0.29166667	2.03E-06	7.61E-04
GO:0030020	extracellular matrix structural constituent conferring tensile strength	molecular_function	structural molecule activity	8	573	39	21691	0.20512821	6.75E-06	0.00168882
GO:0005216	ion channel activity	molecular_function	transporter activity	17	573	194	21691	0.08762887	1.66E-05	0.00312373
GO:0005509	calcium ion binding	molecular_function	binding	41	573	790	21691	0.05189873	3.33E-05	0.00500473
GO:0001968	fibronectin binding	molecular_function	binding	6	573	32	21691	0.1875	1.67E-04	0.02091126
GO:0005104	fibroblast growth factor receptor binding	molecular_function	binding	5	573	28	21691	0.17857143	7.51E-04	0.08054993
GO:0008201	heparin binding	molecular_function	binding	13	573	179	21691	0.0726257	9.79E-04	0.08168177

GO:0008237	metallopeptidase activity	molecular_function	catalytic activity	13	573	178	21691	0.07303371	9.30E-04	0.08168177
GO:0005244	voltage-gated ion channel activity	molecular_function	transporter activity	11	573	142	21691	0.07746479	0.00140313	0.1000006
GO:0007155	cell adhesion	biological_process	biological adhesion	38	582	587	22257	0.06473595	3.20E-07	8.13E-04
GO:0006811	ion transport	biological_process	localization	37	582	611	22257	0.06055646	2.24E-06	0.00272002
GO:0030198	extracellular matrix organization	biological_process	cellular process	15	582	143	22257	0.1048951	5.36E-06	0.00272002
GO:0034097	response to cytokine	biological_process	response to stimulus	12	582	89	22257	0.13483146	3.46E-06	0.00272002
GO:0034220	ion transmembrane transport	biological_process	localization	18	582	196	22257	0.09183673	4.29E-06	0.00272002
GO:0042904	9-cis-retinoic acid biosynthetic process	biological_process	metabolic process	4	582	6	22257	0.66666667	6.66E-06	0.00281505
GO:0001503	ossification	biological_process	multicellular organismal process	12	582	103	22257	0.11650485	1.60E-05	0.0058064
GO:0030178	negative regulation of Wnt signaling pathway	biological_process	regulation of biological process	9	582	61	22257	0.14754098	2.77E-05	0.00879905
GO:0009617	response to bacterium	biological_process	response to stimulus	17	582	209	22257	0.08133971	3.82E-05	0.01077464
GO:0006953	acute-phase response	biological_process	response to stimulus	7	582	39	22257	0.17948718	5.99E-05	0.01520644

Supplementary Table 5. GO analysis of down-regulated mRNAs in GAS of db/db vs. db/m mice. Top 10 significantly changed terms were shown.

GO_C Term ID	GO_C Term Desc	GO_C Term Level1	GO_C Term Level2	Term Candidate Gene Num	Total Candidate Gene Num	Term Gene Num	Total Gene Num	Rich Ratio	*p*-value	FDR
GO:0030018	Z disc	cellular_component	cell	27	1298	157	22482	0.171974522	3.23E-07	2.15E-04
GO:0043218	compact myelin	cellular_component	cell	5	1298	5	22482	1	6.37E-07	2.15E-04
GO:0009986	cell surface	cellular_component	cell	76	1298	745	22482	0.102013423	1.00E-06	2.25E-04
GO:0005737	cytoplasm	cellular_component	cell part	489	1298	7142	22482	0.068468216	1.95E-06	3.30E-04
GO:0005856	cytoskeleton	cellular_component	organelle	113	1298	1272	22482	0.088836478	2.84E-06	3.83E-04
GO:0014704	intercalated disc	cellular_component	cell junction	14	1298	60	22482	0.233333333	6.05E-06	6.07E-04
GO:0020005	symbiont-containing vacuole membrane	cellular_component	extracellular region part	6	1298	10	22482	0.6	6.29E-06	6.07E-04
GO:0030016	myofibril	cellular_component	organelle	12	1298	48	22482	0.25	1.31E-05	8.89E-04
GO:0031430	M band	cellular_component	organelle	10	1298	33	22482	0.303030303	1.08E-05	8.89E-04

GO:0097512	cardiac myofibril	cellular_component	organelle	6	1298	11	22482	0.545454545	1.32E-05	8.89E-04
GO:0003779	actin binding	molecular_function	binding	51	1280	392	21691	0.130102041	9.62E-08	1.22E-04
GO:0000166	nucleotide binding	molecular_function	binding	154	1280	1749	21691	0.088050314	2.50E-07	1.58E-04
GO:0003924	GTPase activity	molecular_function	catalytic activity	43	1280	342	21691	0.125730994	2.38E-06	8.33E-04
GO:0042803	protein homodimerization activity	molecular_function	binding	98	1280	1039	21691	0.094321463	2.64E-06	8.33E-04
GO:0003774	motor activity	molecular_function	catalytic activity	21	1280	123	21691	0.170731707	1.00E-05	0.00253456
GO:0001730	2'-5'-oligoadenylate synthetase activity	molecular_function	catalytic activity	6	1280	11	21691	0.545454545	1.49E-05	0.00314103
GO:0005525	GTP binding	molecular_function	binding	48	1280	439	21691	0.109339408	2.92E-05	0.00409576
GO:0015293	symporter activity	molecular_function	transporter activity	19	1280	112	21691	0.169642857	2.87E-05	0.00409576
GO:0019911	structural constituent of myelin sheath	molecular_function	structural molecule activity	6	1280	12	21691	0.5	2.83E-05	0.00409576
GO:0003785	actin monomer binding	molecular_function	binding	9	1280	30	21691	0.3	3.90E-05	0.00492762
GO:0002376	immune system process	biological_process	immune system process	66	1285	457	22257	0.144420131	5.43E-12	1.16E-08
GO:0009615	response to virus	biological_process	response to stimulus	26	1285	90	22257	0.288888889	3.98E-12	1.16E-08
GO:0009617	response to bacterium	biological_process	response to stimulus	35	1285	209	22257	0.167464115	1.24E-08	1.70E-05
GO:0051607	defense response to virus	biological_process	multi-organism process	35	1285	211	22257	0.165876777	1.59E-08	1.70E-05
GO:0035458	cellular response to interferon-beta	biological_process	response to stimulus	18	1285	66	22257	0.272727273	2.19E-08	1.87E-05
GO:0006936	muscle contraction	biological_process	multicellular organismal process	15	1285	55	22257	0.272727273	3.25E-07	2.31E-04
GO:0045071	negative regulation of viral genome replication	biological_process	regulation of biological process	13	1285	46	22257	0.282608696	1.27E-06	7.73E-04
GO:0042832	defense response to protozoan	biological_process	response to stimulus	11	1285	34	22257	0.323529412	1.90E-06	0.00101293
GO:0003009	skeletal muscle contraction	biological_process	multicellular organismal process	10	1285	30	22257	0.333333333	4.12E-06	0.00195473
GO:0042552	myelination	biological_process	developmental process	14	1285	59	22257	0.237288136	4.89E-06	0.00208798

Supplementary Table 6. Transcription factors were enriched within differentially expressed mRNAs

Gene ID	Gene Symbol	Type	Description	TF Term
107503	Atf5	mRNA	Atf5 activating transcription factor 5	TF_bZIP///bZIP self-build
109575	Tbx10	mRNA	Tbx10 t-box 10	T-box///T-box refers to a group of transcription factors involved in limb and heart development.
11910	Atf3	mRNA	Atf3 activating transcription factor 3	TF_bZIP///bZIP self-build
12394	Runx1	mRNA	Runx1 runt related transcription factor 1	Runt///Runt domain is an evolutionary conserved protein domain.
12399	Runx3	mRNA	Runx3 runt related transcription factor 3	Runt///Runt domain is an evolutionary conserved protein domain.
13983	Esr2	mRNA	Esr2 estrogen receptor 2 (beta)	ESR-like/// zf-C4 self-build
14281	Fos	mRNA	Fos FBJ osteosarcoma oncogene	TF_bZIP///bZIP self-build
14282	Fosb	mRNA	Fosb FBJ osteosarcoma oncogene B	TF_bZIP///bZIP self-build
14283	Fosl1	mRNA	Fosl1 fos-like antigen 1	TF_bZIP///bZIP self-build
15370	Nr4a1	mRNA	Nr4a1 nuclear receptor subfamily 4, group A, member 1	NGFIB-like///zf-C4 self-build
16364	Irf4	mRNA	Irf4 interferon regulatory factor 4	IRF///Interferon regulatory factors are proteins which regulate transcription of interferons.
16391	Irf9	mRNA	Irf9 interferon regulatory factor 9	IRF///Interferon regulatory factors are proteins which regulate transcription of interferons.
16476	Jun	mRNA	Jun jun proto-oncogene	TF_bZIP///bZIP self-build
16477	Junb	mRNA	Junb jun B proto-oncogene	TF_bZIP///bZIP self-build
16658	Mafb	mRNA	Mafb v-maf musculoaponeurotic fibrosarcoma oncogene family, protein B (avian)	TF_bZIP///bZIP self-build
17133	Maff	mRNA	Maff v-maf musculoaponeurotic fibrosarcoma oncogene family, protein F (avian)	TF_bZIP///bZIP self-build

17135	Mafk	mRNA	Mafk v-maf musculoaponeurotic fibrosarcoma oncogene family, protein K (avian)	TF_bZIP///bZIP self-build
18124	Nr4a3	mRNA	Nr4a3 nuclear receptor subfamily 4, group A, member 3	NGFIB-like///zf-C4 self-build
18227	Nr4a2	mRNA	Nr4a2 nuclear receptor subfamily 4, group A, member 2	NGFIB-like///zf-C4 self-build
21380	Tbx1	mRNA	Tbx1 T-box 1	T-box///T-box refers to a group of transcription factors involved in limb and heart development.
21386	Tbx3	mRNA	Tbx3 T-box 3	T-box///T-box refers to a group of transcription factors involved in limb and heart development.
21387	Tbx4	mRNA	Tbx4 T-box 4	T-box///T-box refers to a group of transcription factors involved in limb and heart development.
22061	Trp63	mRNA	Trp63 transformation related protein 63	P53///P53 DNA-binding domain
22062	Trp73	mRNA	Trp73 transformation related protein 73	P53///P53 DNA-binding domain
26380	Esrrb	mRNA	Esrrb estrogen related receptor, beta	ESR-like/// zf-C4 self-build
26381	Esrrg	mRNA	Esrrg estrogen-related receptor gamma	ESR-like/// zf-C4 self-build
27056	Irf5	mRNA	Irf5 interferon regulatory factor 5	IRF///Interferon regulatory factors are proteins which regulate transcription of interferons.
378435	Mafa	mRNA	Mafa v-maf musculoaponeurotic fibrosarcoma oncogene family, protein A (avian)	TF_bZIP///bZIP self-build
54123	Irf7	mRNA	Irf7 interferon regulatory factor 7	IRF///Interferon regulatory factors are proteins which regulate transcription of interferons.

Supplementary Table 7. KEGG pathway enrichment up-regulated mRNAs with top 10 Enrichment score.

KEGG Pathway Term ID	KEGG Pathway Term	count	Rich Ratio	*p*-value	FDR
pathway: 04080	Neuroactive ligand-receptor interaction	26	0.08469055	2.67E-07	5.45E-05
pathway: 04024	cAMP signaling pathway	16	0.07655502	1.93E-04	0.01348491
pathway: 04512	ECM-receptor interaction	10	0.10869565	1.98E-04	0.01348491
pathway: 04151	PI3K-Akt signaling pathway	23	0.05707196	6.63E-04	0.03379629
pathway: 04923	Regulation of lipolysis in adipocytes	7	0.12068966	9.70E-04	0.03956414
pathway: 04974	Protein digestion and absorption	9	0.09183674	0.00141169	0.04799743
pathway: 04060	Cytokine-cytokine receptor interaction	18	0.05642633	0.0028831	0.08231081
pathway: 04964	Proximal tubule bicarbonate reclamation	4	0.17391304	0.00322788	0.08231081
pathway: 04971	Gastric acid secretion	7	0.09210526	0.00465304	0.1054688
pathway: 00830	Retinol metabolism	8	0.08080808	0.00566522	0.1155705

Supplementary Table 8. KEGG pathway enrichment down-regulated mRNAs with top 10 Enrichment score.

KEGG Pathway Term ID	KEGG Pathway Term	count	Rich Ratio	*p*-value	FDR
pathway: 04260	Cardiac muscle contraction	18	0.227848101	1.49E-06	3.71E-04
pathway: 04514	Cell adhesion molecules (CAMs)	29	0.138755981	5.02E-05	0.004678135
pathway: 04621	NOD-like receptor signaling pathway	28	0.137931034	7.52E-05	0.004678135
pathway: 04668	TNF signaling pathway	22	0.156028369	7.05E-05	0.004678135
pathway: 00330	Arginine and proline metabolism	12	0.181818182	7.81E-04	0.038879672
pathway: 00480	Glutathione metabolism	13	0.158536585	0.001800797	0.058268988
pathway: 04060	Cytokine-cytokine receptor interaction	34	0.106583072	0.001872096	0.058268988
pathway: 04612	Antigen processing and presentation	18	0.135338346	0.001751373	0.058268988
pathway: 04261	Adrenergic signaling in cardiomyocytes	20	0.126582278	0.002269368	0.062785848
pathway: 00960	Tropane, piperidine and pyridine alkaloid biosynthesis	3	0.5	0.004377338	0.108995716

Supplementary Table 9. Gene set enrichment analysis of differentially expressed mRNAs in GAS of *db/db* vs. *db/m* mice

KEGG pathway term ID	KEGG Pathway Term	Count	Enrichment score (ES)	Normalized enrichment score (NES)	*p*-value	FDR
**Path: 00190**	Oxidative phosphorylation	127	-0.5526848	-2.0429156	0	0.00222351
**Path: 00330**	Arginine and proline metabolism	54	-0.5889819	-1.8983037	0	0.01518323
**Path: 04621**	NOD-like receptor signaling pathway	165	-0.49058524	-1.8674083	0	0.01310986
**Path: 04260**	Cardiac muscle contraction	73	-0.53326494	-1.8339995	0	0.01554834
**Path: 03040**	Spliceosome	145	-0.48090827	-1.7948809	0	0.01919736
**Path: 04668**	TNF signaling pathway	121	-0.47786793	-1.7660106	0	0.02615792
**Path: 04714**	Thermogenesis	224	-0.44887698	-1.760888	0	0.02452575
**Path: 04215**	Apoptosis - multiple species	32	-0.58779675	-1.7351348	0.001472754	0.03038663
**Path: 04612**	Antigen processing and presentation	92	-0.48597893	-1.7295831	0	0.02789833
**Path: 00970**	Aminoacyl-tRNA biosynthesis	44	-0.55156153	-1.7230982	0.00270636	0.02693685
**Path: 00520**	Amino sugar and nucleotide sugar metabolism	52	-0.5233406	-1.7163136	0.001347709	0.02562705
**Path: 03008**	Ribosome biogenesis in eukaryotes	78	-0.49748105	-1.710581	0	0.0262608
**Path: 00620**	Pyruvate metabolism	38	-0.55128986	-1.7048439	0	0.02579342
**Path: 00270**	Cysteine and methionine metabolism	45	-0.5256719	-1.674743	0.002688172	0.03695324
**Path: 04622**	RIG-I-like receptor signaling pathway	56	-0.5064118	-1.6657387	0.009383378	0.03925965
**Path: 01110**	Biosynthesis of secondary metabolites	408	-0.4016611	-1.64127	0	0.04969784
**Path: 00020**	Citrate cycle (TCA cycle)	33	-0.5588199	-1.6380706	0.007062147	0.04857261
**Path: 00760**	Nicotinate and nicotinamide metabolism	37	-0.52634275	-1.6229776	0.009873061	0.05352934

**Path: 00240**	Pyrimidine metabolism	103	-0.44775844	-1.622756	0.00239521	0.05083772
**Path: 01120**	Microbial metabolism in diverse environments	197	-0.41644928	-1.6215492	0	0.04891422
**Path: 00052**	Galactose metabolism	34	-0.5362767	-1.6123178	0.015602837	0.05217607
**Path: 04141**	Protein processing in endoplasmic reticulum	175	-0.4203752	-1.6065907	0	0.05313872
**Path: 03020**	RNA polymerase	30	-0.54538834	-1.6059985	0.008275862	0.05111904
**Path: 04210**	Apoptosis	139	-0.43250751	-1.6047664	0	0.04955835
**Path: 03030**	DNA replication	35	-0.52853376	-1.603016	0.016574586	0.04829614
**Path: 00790**	Folate biosynthesis	25	-0.5647241	-1.5977005	0.018284107	0.04892057
**Path: 04217**	Necroptosis	168	-0.41426623	-1.5951003	0.001132503	0.04857654
**Path: 00630**	Glyoxylate and dicarboxylate metabolism	31	-0.53893936	-1.5940472	0.008720931	0.04759575
**Path: 01230**	Biosynthesis of amino acids	88	-0.4495537	-1.5777572	0	0.05520424
**Path: 01130**	Biosynthesis of antibiotics	237	-0.4020137	-1.57719	0	0.05358679
**Path: 01200**	Carbon metabolism	138	-0.4250244	-1.5748466	0.001187649	0.05277475
**Path: 00120**	Primary bile acid biosynthesis	15	-0.63615996	-1.5686022	0.020833334	0.05487995
**Path: 04216**	Ferroptosis	44	-0.4959733	-1.5655997	0.012096774	0.05520907
**Path: 00561**	Glycerolipid metabolism	67	-0.46571037	-1.5636723	0.00794702	0.05472251
**Path: 04623**	Cytosolic DNA-sensing pathway	52	-0.483717	-1.5538176	0.01321004	0.05908325
**Path: 03050**	Proteasome	49	-0.4789619	-1.5368923	0.010596027	0.06931855
**Path: 00670**	One carbon pool by folate	20	-0.57616717	-1.529518	0.030837005	0.07297418
**Path: 00600**	Sphingolipid metabolism	47	-0.47145516	-1.5163796	0.013175231	0.07978042
**Path: 04144**	Endocytosis	277	-0.37838146	-1.4988393	0	0.09153216
**Path: 00983**	Drug metabolism - other enzymes	80	-0.4240861	-1.4832975	0.014084507	0.10244641
**Path: 00720**	Carbon fixation pathways in prokaryotes	16	-0.58658737	-1.4786525	0.042682927	0.10412729
**Path: 00230**	Purine metabolism	176	-0.38347492	-1.4747382	0.003409091	0.10543973
**Path: 04380**	Osteoclast differentiation	129	-0.39830652	-1.4646047	0.012048192	0.11283693

**Path: 04212**	Longevity regulating pathway - worm	73	-0.42626804	-1.4621549	0.013871375	0.11319479
**Path: 00564**	Glycerophospholipid metabolism	105	-0.40026918	-1.4492073	0.019680196	0.1236824
**Path: 04066**	HIF-1 signaling pathway	108	-0.39681992	-1.4367079	0.017199017	0.1322489
**Path: 04620**	Toll-like receptor signaling pathway	90	-0.40204182	-1.4335115	0.030150754	0.13308004
**Path: 04630**	Jak-STAT signaling pathway	139	-0.38611218	-1.4329159	0.014285714	0.13118261
**Path: 04514**	Cell adhesion molecules (CAMs)	165	-0.3714898	-1.4254291	0.009174312	0.13814448
**Path: 00280**	Valine, leucine and isoleucine degradation	56	-0.4284441	-1.4168553	0.04953146	0.14301506
**Path: 04137**	Mitophagy - animal	67	-0.40939978	-1.389222	0.030534351	0.17517814
**Path: 00480**	Glutathione metabolism	67	-0.41029608	-1.3777372	0.03529412	0.18587226
**Path: 00010**	Glycolysis / Gluconeogenesis	75	-0.40324086	-1.3750738	0.04865557	0.18318556
**Path: 04625**	C-type lectin receptor signaling pathway	109	-0.3726755	-1.361083	0.043156598	0.1979647
**Path: 04071**	Sphingolipid signaling pathway	128	-0.36472544	-1.3609551	0.030805686	0.19488622
**Path: 04145**	Phagosome	174	-0.35446945	-1.3420619	0.023809524	0.21662267
**Path: 03013**	RNA transport	176	-0.3414098	-1.302725	0.03686636	0.25030002
**Path: 03010**	Ribosome	175	-0.33895674	-1.2990037	0.04084014	0.24999419
**Path: 04924**	Renin secretion	70	0.34984162	1.4207784	0.026548672	0.55237496
**Path: 04080**	Neuroactive ligand-receptor interaction	237	0.30620188	1.4746653	0.012987013	0.7719984

Supplementary Table 10. Top 20 differential expressed lncRNAs in GAS of db/db vs. db/m (FDR <0.05).

Gene ID	Gene Symbol	Log_2 FC	*p*-value	FDR	style
74184	2310065F04Rik	0.95011817	0	0	Up
442820	D830005E20Rik	0.89990918	6.05E-165	4.10E-163	Up
320092	E030003E18Rik	1.33826233	4.05E-114	2.03E-112	Up
73339	1700047G03Rik	6.34827874	3.25E-97	1.44E-95	Up
105246838	Gm42060	0.66616603	4.67E-66	1.57E-64	Up
668215	Hectd2os	1.43472666	8.58E-64	2.78E-62	Up
319940	Sorbs2os	0.86407456	8.87E-62	2.80E-60	Up
102635783	Gm11266	0.97542592	1.61E-58	4.84E-57	Up
102637887	Gm34583	4.06953792	1.16E-51	3.13E-50	Up
100038752	Gm10825	1.76567405	5.21E-47	1.31E-45	Up
102635293	Gm32670	0.72934813	2.88E-46	7.11E-45	Up
105246464	Gm41750	0.82144209	1.52E-44	3.65E-43	Up
434510	Gm5627	4.81867823	1.23E-40	2.79E-39	Up
73504	1700071M16Rik	1.60902261	1.25E-40	2.83E-39	Up
102635277	Gm32658	1.14068485	2.92E-38	6.26E-37	Up
78849	B430010I23Rik	0.69583653	4.34E-33	8.24E-32	Up
105246216	Gm41530	0.99684193	2.69E-32	5.00E-31	Up
102632040	Gm30215	1.7171398	2.79E-31	5.10E-30	Up
102633155	Gm31054	0.63657404	9.36E-31	1.69E-29	Up
105247135	Gm12514	2.94420912	1.56E-29	2.73E-28	Up
14955	H19	-0.7616798	0	0	Down
232685	Lncpint	-0.6722016	0	0	Down
102639019	Gm35438	-1.8984431	0	0	Down
102637598	Gm34365	-0.7637262	1.90E-283	2.13E-281	Down
402730	Foxo6os	-2.5437152	4.21E-129	2.34E-127	Down
434353	A330074K22Rik	-1.1297722	3.84E-117	1.97E-115	Down
100043424	Gm14005	-0.8456478	3.87E-96	1.70E-94	Down
100504114	Gm16907	-1.6343649	1.34E-89	5.51E-88	Down
102640269	Gm36380	-1.8214929	1.04E-65	3.50E-64	Down
320846	A530058N18Rik	-2.3550792	7.22E-65	2.38E-63	Down
102634407	Gm24576	-0.9369906	1.42E-58	4.26E-57	Down
102634685	Gm17197	-1.0761613	3.49E-57	1.03E-55	Down
102635346	Gm32709	-0.8480269	1.28E-55	3.66E-54	Down
105246964	Gm42154	-2.1359105	4.10E-55	1.16E-53	Down
102635269	Gm32652	-1.7465774	4.60E-51	1.24E-49	Down
433374	Gm20743	-1.5756863	3.67E-49	9.60E-48	Down
100504641	1700101I11Rik	-1.8252316	2.37E-47	5.97E-46	Down
102634786	Gm32287	-1.26958	2.79E-46	6.89E-45	Down
100503613	5031434C07Rik	-1.26248	3.41E-46	8.40E-45	Down
77920	A330102I10Rik	-1.9847906	2.09E-44	5.01E-43	Down

Note： lncRNAs: long non-coding RNAs. FC: fold change; FDR: false discovery rate.

Supplementary Table 11. RT-qPCR Primers

Gene name	Gene type	primers
**Gm20743**	lncRNA	Forward: GCAGAACTGGGACTTGGAAAGGG<br>Reverse: GAGTGTGTGGTGGAGGCTGTTTAC
**Gm36131**	lncRNA	Forward: GCCTGTGTCACCTCTGCTGAATAG<br>Reverse: TGGCGTTCCTGAGAGACCTTAGAG
**A330074K22Rik**	lncRNA	Forward: TGGCTCGGGCTGTCTGAAGG<br>Reverse: GGTCTGATGATTCTGGCACTAGCG
**Gm35438**	lncRNA	Forward: CCAAGTGACCCAACAACCCAAGG<br>Reverse: GGCTCGTTCACACTCGCTCTTC
**Gm31814**	lncRNA	Forward: GTTGTCAGGCTCAGTGGTCACATC<br>Reverse: GTATTTCCTTCTGGTGGCGAGTCC
**1700047G03Rik**	lncRNA	Forward: GTTCCCTCGGCTTTCTGTCCATTC<br>Reverse: AGGCAGACGGACTCTATGACTTCG
**FBOX32**	mRNA	Forward: GAATGCCTGTTTGCCCCTGGAG<br>Reverse: TGTAGGGACTCACCGTAGCG
**Mstn**	mRNA	Forward: GAAAAAGAGGGGCTGTGTAATG<br>Reverse: CATCTTTGCTGATGTTAGGAGC
**TRIM63**	mRNA	Forward: TCATCCTGCCCTGCC AACA<br>Reverse: AGTAGGACGGGACGGTTGT
**ATPb1**	mRNA	Forward: GAAGGACGACATGATTTTCGAG<br>Reverse: CACTCGGTTGAGCTTGATAATG
**Gclc**	mRNA	Forward: TGTCCGAGTTCAATACAGTTGA<br>Reverse: ACAGCCTAATCTGGGAAATGAA
**Gclm**	mRNA	Forward: CTTGGAGCATTTACAGCCTTAC<br>Reverse: GTGAGTCAGTAGCTGTATGTCA
**GPX4**	mRNA	Forward: GCCTCGAGATGTGTGCATCCCGCGATGA<br>Reverse: GCGGATCCCTAGAGATAGCACGGCAGGT
**Tnnc1**	mRNA	Forward: GATTGACGAAGTAGACGAGGAT<br>Reverse: GTTTTTGTCAAACATGCGGAAG
**Tpm3**	mRNA	Forward: GAAGAATGTCACCAACAACCTC<br>Reverse: GTCTCTGCCTCCTTGAGTTTAT
**ACSL3**	mRNA	Forward: CGGAAATCATGGATCGGATCTA<br>Reverse: GTGGAGTACTACACCCTTTTGA
**DGAT2**	mRNA	Forward: GAAGTCAGCAAGAAGTTTCCTG<br>Reverse: CACCACGATGATGATAGCATTG
**ACTB**	mRNA	Forward: CTACCTCATGAAGATCCTGACC<br>Reverse: CACAGCTTCTCTTTGATGTCAC

Note： RT-qPCR: Real-Time Quantitative PCR; lncRNAs: long non-coding RNAs.
