# Supplementary material for: The Whole-transcriptome Landscape of Diabetes-related Sarcopenia Reveals the Specific Function of Novel lncRNA Gm20743
Source: Commun Biol. 2022 Aug 1;5:774. doi: 10.1038/s42003-022-03728-8 (PMC9343400; doi:10.1038/s42003-022-03728-8)
Supplement: Supplementary file 2 — Description of Additional Supplementary Files [file 42003_2022_3728_MOESM2_ESM.pdf]

Description of Additional Supplementary Files

File name: Supplementary Data 1

Description: Upregulated mRNAs in GAS of *db/db* vs. *db/m* mice.

File name: Supplementary Data 2

Description: Downregulated mRNAs in GAS of *db/db* vs. *db/m* mice.

File name: Supplementary Data 3

Description: Upregulated lncRNAs in GAS of *db/db* vs. *db/m* mice.

File name: Supplementary Data 4

Description: Downregulated lncRNAs in GAS of *db/db* vs. *db/m* mice.

File name: Supplementary Data 5

Description: All source data used for generating graphs and charts in main and supplementary figures.
