# Supplementary material for: The Whole-transcriptome Landscape of Diabetes-related Sarcopenia Reveals the Specific Function of Novel lncRNA Gm20743
Source: Commun Biol. 2022 Aug 1;5:774. doi: 10.1038/s42003-022-03728-8 (PMC9343400; doi:10.1038/s42003-022-03728-8)
Supplement: Supplementary file 8 — Reporting-summary [file 42003_2022_3728_MOESM8_ESM.pdf]

nature portfolio

Corresponding author(s): Qian Xiao

Last updated by author(s): Jun 20, 2022

Reporting Summary

Nature Portfolio wishes to improve the reproducibility of the work that we publish. This form provides structure for consistency and transparency in reporting. For further information on Nature Portfolio policies, see our Editorial Policies and the Editorial Policy Checklist.

Statistics

For all statistical analyses, confirm that the following items are present in the figure legend, table legend, main text, or Methods section.

n/a	Confirmed	
☐	☒	The exact sample size (*n*) for each experimental group/condition, given as a discrete number and unit of measurement
☐	☒	A statement on whether measurements were taken from distinct samples or whether the same sample was measured repeatedly
☐	☒	The statistical test(s) used AND whether they are one- or two-sided<br>*Only common tests should be described solely by name; describe more complex techniques in the Methods section.*
☒	☐	A description of all covariates tested
☒	☐	A description of any assumptions or corrections, such as tests of normality and adjustment for multiple comparisons
☐	☒	A full description of the statistical parameters including central tendency (e.g. means) or other basic estimates (e.g. regression coefficient) AND variation (e.g. standard deviation) or associated estimates of uncertainty (e.g. confidence intervals)
☒	☐	For null hypothesis testing, the test statistic (e.g. *F*, *t*, *r*) with confidence intervals, effect sizes, degrees of freedom and *P* value noted<br>*Give P values as exact values whenever suitable.*
☒	☐	For Bayesian analysis, information on the choice of priors and Markov chain Monte Carlo settings
☒	☐	For hierarchical and complex designs, identification of the appropriate level for tests and full reporting of outcomes
☐	☒	Estimates of effect sizes (e.g. Cohen's *d*, Pearson's *r*), indicating how they were calculated

Our web collection on statistics for biologists contains articles on many of the points above.

Software and code

Policy information about availability of computer code

Data collection	No software was used.
Data analysis	The data analysis conducted in this paper was done using open source statistical tools and published methods, as described in the text.

For manuscripts utilizing custom algorithms or software that are central to the research but not yet described in published literature, software must be made available to editors and reviewers. We strongly encourage code deposition in a community repository (e.g. GitHub). See the Nature Portfolio guidelines for submitting code & software for further information.

Data

Policy information about availability of data

All manuscripts must include a data availability statement. This statement should provide the following information, where applicable:

- Accession codes, unique identifiers, or web links for publicly available datasets
- A description of any restrictions on data availability
- For clinical datasets or third party data, please ensure that the statement adheres to our policy

A data availability statement is provided in the manuscript.

Human research participants

Policy information about studies involving human research participants and Sex and Gender in Research.

Reporting on sex and gender	No clinical data and human participants in this paper.
Population characteristics	No clinical data and human participants in this paper.
Recruitment	No clinical data and human participants in this paper.
Ethics oversight	No clinical data and human participants in this paper.

Note that full information on the approval of the study protocol must also be provided in the manuscript.

Field-specific reporting

Please select the one below that is the best fit for your research. If you are not sure, read the appropriate sections before making your selection.

☒ Life sciences ☐ Behavioural & social sciences ☐ Ecological, evolutionary & environmental sciences

For a reference copy of the document with all sections, see nature.com/documents/nr-reporting-summary-flat.pdf

Life sciences study design

All studies must disclose on these points even when the disclosure is negative.

Sample size	Animal experimental group had a replicate number of 10 (10 db/m and 10 db/db) mice. In addition to RNA-sequencing, mice were required for tests including grip strength, body composition analysis, histopathology, electron microscopy, and RT-qPCR. To ensure the relative accuracy of the sequencing results, we selected a relatively suitable number of biological replicates (n=3) for RNA-seq. We performed RT-qPCR verification on the gene expression in gastrocnemius muscle(n=5). The sample size was sufficient to obtain acceptable error bars and draw a conclusion about the understanding of lncRNAs in diabetes-induces sarcopenia.
Data exclusions	No data is exclude from this paper. For the comparison to RT-qPCR analysis, the CT values greater than 35 were excluded from the final analysis.
Replication	Considering all the measurements, a standard deviation and uncertainties were calculated as described in this study. All attempts at replication were successful.
Randomization	We used db/db mice and their wildtype littlemate mice as purchased from SPF Biotechnology Company (Beijing, China).
Blinding	In terms of blinding the study, the function of Gm20743 was determined by knock-down and over-expression experiments by the unknown grouping researchers.

Reporting for specific materials, systems and methods

We require information from authors about some types of materials, experimental systems and methods used in many studies. Here, indicate whether each material, system or method listed is relevant to your study. If you are not sure if a list item applies to your research, read the appropriate section before selecting a response.

Materials & experimental systems

n/a	Involved in the study	
☐	☒	Antibodies
☐	☒	Eukaryotic cell lines
☒	☐	Palaeontology and archaeology
☐	☒	Animals and other organisms
☒	☐	Clinical data
☒	☐	Dual use research of concern

Methods

n/a	Involved in the study	
☒	☐	ChIP-seq
☒	☐	Flow cytometry
☒	☐	MRI-based neuroimaging

Antibodies

Antibodies used	The antibody of myosin heavy chain was purchase from Sigma-Aldrich, catalog number is M1570.
Validation	The antibody of myosin heavy chain was used for immunofluorescence.

Eukaryotic cell lines

Policy information about cell lines and Sex and Gender in Research

Cell line source(s)	Mouse C2C12 cells which purchases from ATCC were used in this study.
Authentication	Mouse C2C12 cell line was authenticated when we purchase the cell line.
Mycoplasma contamination	Mouse C2C12 cell was tested negative for mycoplasma contamination.
Commonly misidentified lines (See ICLAC register)	There is no commonly misidentified cell line in this study.

Animals and other research organisms

Policy information about studies involving animals; ARRIVE guidelines recommended for reporting animal research, and Sex and Gender in Research

Laboratory animals	15-week-old male db/db mice (C57BLKS- Leprdb/db diabetic mice) and db/m male wild-type littermates (C57BLKS- Leprdb/m mice) were used in this study.
Wild animals	No wild animals in the study.
Reporting on sex	There are only male mice in this study. Considering the possible protective factors of estrogen in female mice, we only selected male mice for the study.
Field-collected samples	Mice were housed in a pathogen-free environment with a temperature of 22 ± 2°C, humidity of 60% and 12-hour light/12-hour dark cycles.
Ethics oversight	All animal experiments were conducted in accordance with standard animal care protocols. This study was approved by the Animal Ethics Committee of The First Affiliated Hospital of Chongqing Medical University.

Note that full information on the approval of the study protocol must also be provided in the manuscript.
